# Masculinization of populations reverses sex differences in fertility

**DOI:** 10.1073/pnas.2533317123

**Published:** 2026-04-20

**Authors:** Henrik-Alexander Schubert, Thomas Spoorenberg, Christian Dudel, Vegard Fykse Skirbekk

**Affiliations:** ^a^Department of Social Demography, Max-Planck Institute for Demographic Research, Mecklenburg-Vorpommern, Rostock 18057, Germany; ^b^Max Planck–University of Helsinki Center for Social Inequalities in Population Health, Helsinki 00100, Finland; ^c^Department of Economic and Social Affairs, UN Population Division, New York, NY 10017; ^d^Federal Institute for Population Research, Wiesbaden, Germany; ^e^Centre for Fertility and Health, Norwegian Institute for Public Health, Oslo 0473, Norway; ^f^Department of Psychology, University of Oslo, Oslo 0373, Norway

**Keywords:** demography, sex-selective abortion, male fertility, marriage markets, sex difference

## Abstract

Reproductive-age populations show a growing male surplus around the globe as a consequence of declining mortality, narrowing sex differences in mortality, and sex-selective abortions in some countries. Population structures are important determinants of marriage markets and childbearing. In this study, we estimate the past, current, and future difference between the male and the female total fertility rates around the world using an established indirect demographic approach drawing on data from the UN World Population Prospects. Our results indicate a crossover from historically higher male fertility to increasingly higher female fertility, which occurs globally in 2024. This shift is not toward parity, but rather reflects a growing disparity driven by the increasing male surplus at reproductive ages, which exerts downward pressure on male fertility rates relative to those of women. The difference is expected to grow to up to 20% in countries like China and India, where sex-selective abortion has reinforced sex imbalances in population structures. Overall, we highlight the growing sex inequalities in reproduction and call for more research on sex differences in fertility.

## Significance

Human fertility assessments are disproportionally focusing on women’s childbearing. However, narrowing gender gaps in mortality and sex selective abortion contribute to a masculinization of population structures, which might in turn affect male–female fertility differentials. Our analysis shows that over time this has led to a crossover from lower fertility among women to lower fertility among men. We identify 2024 as the year when female global fertility levels first exceeded male fertility. Population projections until 2100 indicate that a return to parity is unlikely. A masculinization of population structures raises barriers for parenting for men with consequences for their social connections, social and economic support.

Fertility is a fundamental demographic process that shapes population structures and profoundly influences individual well-being. Fertility is commonly measured for women but not for men. For instance, statistical offices routinely report the average number of children per woman. Focusing only on women can be misleading in populations with imbalanced population structures, because it does not truly reflect the reproductive behavior of the entire population. While previous studies have demonstrated a strong synchrony between male and female fertility trends globally (1, 2), notable deviations emerge in high-fertility contexts (3) or in populations with marked sex imbalances (2, 4, 5). Nonetheless, the historical evolution and future trajectories of sex disparities in fertility remain poorly understood at the global scale.

Sex differences in fertility arise from two interrelated factors: sex-specific population structures and differential fertility timing. Because fertility is defined as the number of births relative to the population exposed to childbearing, imbalances in the sex ratio of the reproductive population can lead to divergent fertility estimates between sexes. Moreover, men typically exhibit a broader reproductive age window and have children later than women, which can result in higher observed fertility rates among men in young and growing populations (3, 6). These patterns are further modulated by demographic shocks, such as abrupt fertility transitions or mortality crises, that alter the age and sex composition of the population, thereby influencing the observed sex gap in fertility (1, 2, 4, 7).

Sex differences in fertility may indicate imbalanced mating markets in which the more abundant sex faces structural constraints on partnership formation and may affect union composition in terms of the age gap between the partners and the partners’ bargaining power (8–13). Another concern is the effect on fertility and childlessness, as cohorts exposed to sex differences in fertility face a structural constraint to childbearing, potentially leading to increased childlessness among men and women (2, 14, 15). Finally, it is hypothesized that sex differences in partnering and fertility have downstream implications for social and health outcomes, including increased violence and the spread of sexually transmitted diseases, particularly among individuals who are unpartnered and childless (16–22).

This study presents a global analysis of male and female total fertility rates (TFRs). The TFR indicates the average number of children born to a woman or a man by the end of their reproductive period if they were subject to the age-specific fertility rates of a given year. We examine historical trends and future projections of sex differences in fertility across countries and areas, building on a regression-based prediction of the TFR of men, as suggested by keilman et al. (23) and leveraging data from the United Nations World Population Prospects 2024 (24). We also use classic demographic standardization to assess the impact of the population structure on sex differences in fertility, removing the impact of age differences in partnering.

Our findings reveal a striking temporal shift: While male TFRs ($TFR_{m}$) historically exceeded female TFRs ($TFR_{w}$) in most nations, a crossover has occurred in recent decades, with the $TFR_{w}$ now surpassing the $TFR_{m}$ in an increasing number of countries and areas. From 2030 onward, the majority of the world population will be living in countries with significantly (δ≥5%) lower $TFR_{m}$ than the $TFR_{w}$. This reversal reflects underlying changes in population structure driven by sex-specific mortality and sex ratios at birth. By disentangling these demographic forces, we demonstrate how shifts in the sex composition of reproductive-age populations – particularly through differential survival and sex-selective birth patterns – have fundamentally reshaped the sex-specific dynamics of fertility over time.

## Declining Male Fertility

**Fig. 1.**
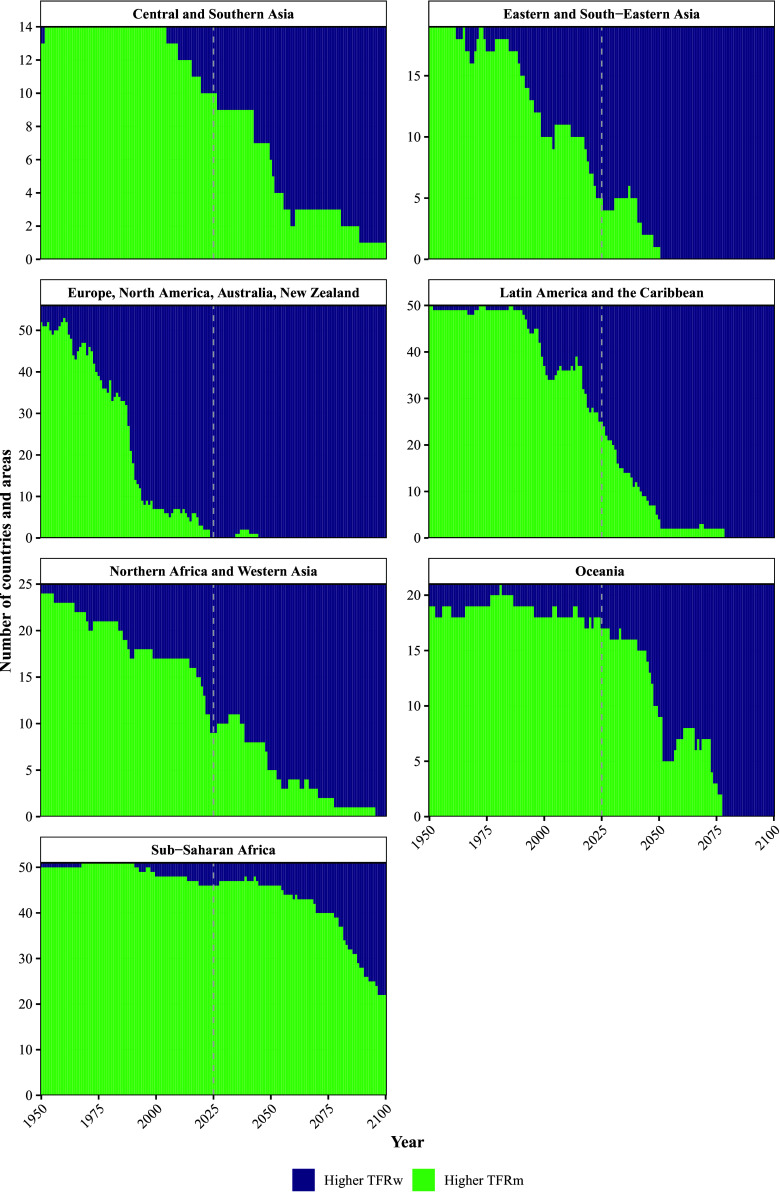
The number of countries and areas with a higher $TFR_{m}$ in green and the number of countries with a higher $TFR_{w}$ in blue in the period between 1950 and 2100 (x-axis) by SDG region.

For the overall transition of populations from a higher $TFR_{m}$ in the past to a higher $TFR_{w}$ in the future, see Fig. 1, which shows a reversal of the sex inequality in reproduction in most parts of the world, with the exception of sub-Saharan Africa. In 1950, the $TFR_{m}$ exceeded the $TFR_{w}$ around the world with 96.2% of the countries and areas showing a higher TFR for men than for women. However, the decline in the $TFR_{m}$ has been steeper than that in the $TFR_{w}$, resulting in a mixed pattern in 2025, with some countries and areas exhibiting a higher $TFR_{m}$ and others a higher $TFR_{w}$. In 2025, 47.5% of countries and areas have higher $TFR_{m}$, but this share is expected to fall sharply to only 9.8% by 2100.

**Fig. 2.**
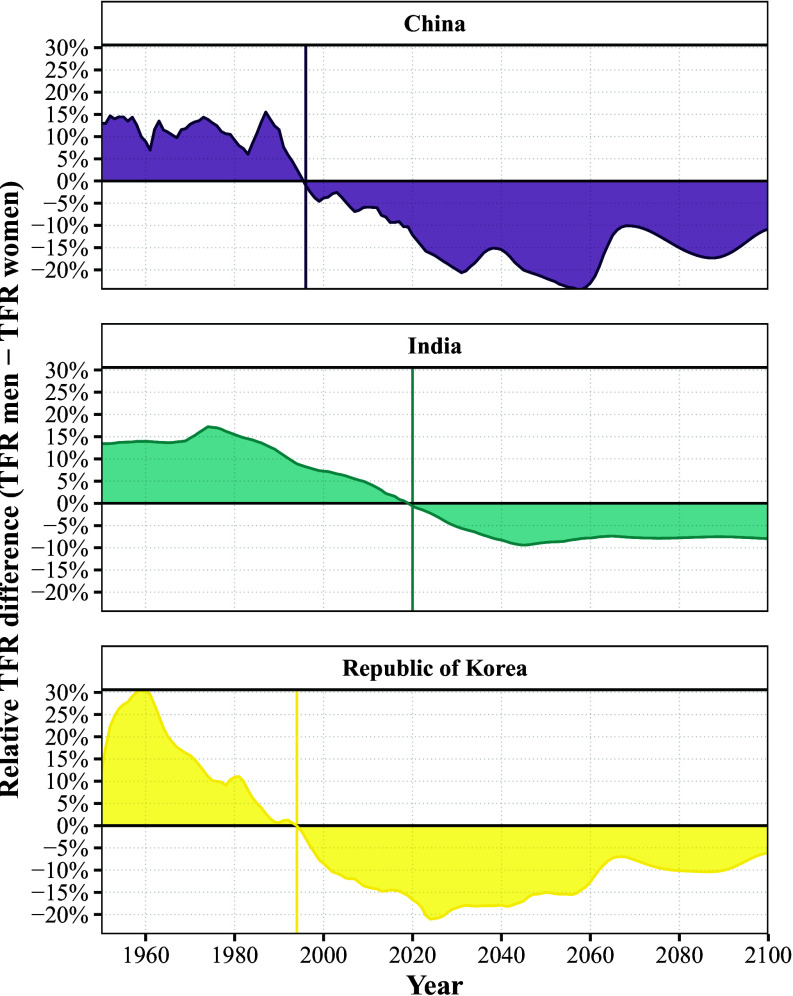
Percentage difference in male to female TFR (y-axis) in the period between 1950 to 2100 (x-axis) using the regression-based approach. Positive values indicate a higher TFR among men and negative values indicate a lower TFR among men. The vertical lines indicate the crossover from a higher male TFR to a higher female TFR.

The difference between the $TFR_{m}$ and the $TFR_{w}$ can be substantial, and ranges from −61.6% (Qatar, 2009) to +131.01% (Turks and Caicos Islands, 1975). While most countries will only fall marginally below equilibrium ($TFR_{m}-TFR_{w}=0$), the majority of the world population will be living in countries with more than 5% lower $TFR_{m}$ than $TFR_{w}$ after 2030. Extreme cases are often found in smaller countries and areas, where a modest change in mortality or migration affecting only one sex can greatly alter the relative size of the male and the female population. However, even in countries and areas with large populations where sex-selective abortion is common, such as China, India, and the Republic of Korea, marked differences between male and female TFRs are observed. Fig. 2 shows the relative difference between the male and the female TFR, revealing that a crossover occurred in China in 1996, in India in 2020, and in the Republic of Korea in 1994. Moreover, these countries will reach the minimum of the relative difference in the 2020s and 2030s, respectively, indicating that sex disparities in fertility are likely to become more pronounced in the near future.

The time point when the $TFR_{w}$ first exceeded the $TFR_{m}$ globally occurred in the year 2024, but the timing of the fertility crossover varies across geographic regions, see Fig. 1. In the majority of European and North American countries, this crossover happened decades ago, mainly in the 1960s and 1970s. In most Latin American countries, the crossover happened in the recent past. The majority of countries in North Africa, East Asia, Oceania, and Central Asia are expected to experience the crossover in the near future. In countries in sub-Saharan Africa, the crossover is expected to occur much farther into the future, with many of these countries not experiencing the crossover before 2100.

We use demographic standardization to show the impact of gender differences in the population on sex differences in fertility in the absence of age gaps between parents (*Materials and methods*). The standardization results corroborate the regression-based results, showing that the male TFR declines relative to the female TFR over time, which indicates that population sex ratios are the main driver of differences between the $TFR_{m}$ and the $TFR_{w}$. The conclusion is supported by a supplementary counterfactual simulation of the change in the TFR ratios (*SI Appendix*, *Decomposition of Drivers of TFR Ratio Change*). However, two noticeable differences emerge between the regression-based and the standardization approaches. First, fewer crossovers occur in the standardization results than in the regression-based results. Second, the sex differences in fertility in the past are weaker and more muted. Both observations may be related to the fact that it is not just sex differences in population structures that drive the sex differences in fertility, but also larger age differences between fathers and mothers and high population growth rates, which offset the impact of male-skewed populations at reproductive ages (3) (*SI Appendix*, Fig. S13).

### Untangling the Demographic Drivers.

**Fig. 3.**
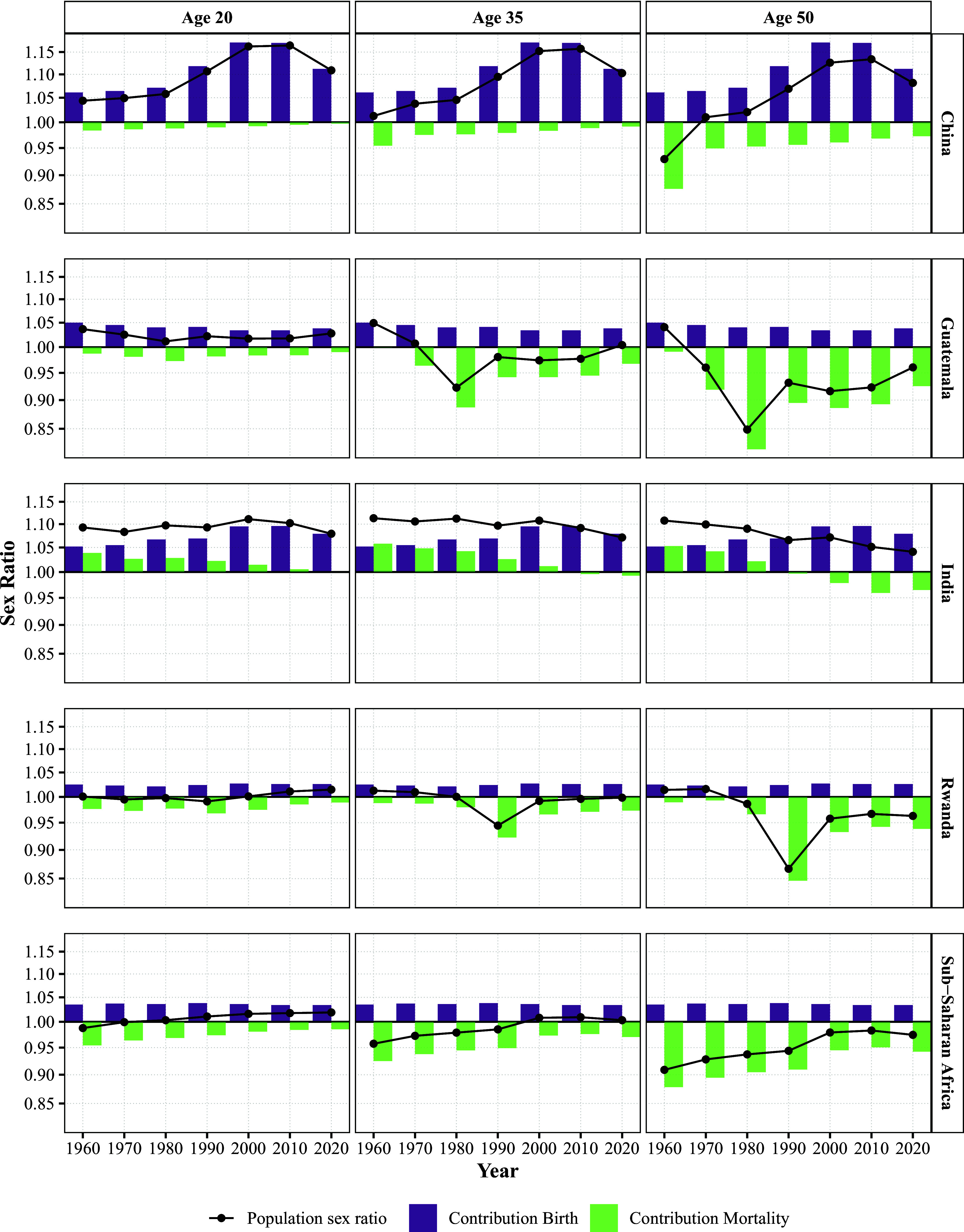
This figure decomposes the period age-specific population sex ratio (black line with dots) into contributions from the sex ratio at birth (purple bars) and sex-differences in mortality (green bars) across countries (vertical panels) and for different ages (horizontal panels). The y-axis displays the population, birth, and mortality sex ratio, and the x-axis displays the corresponding year. Higher values indicate a larger share of men in the population, values of 1 indicate a sex balance, and values below 1 indicate larger share of women in the population.

Fig. 3 illustrates how sex ratios at birth and sex differences in mortality shape the population sex ratios in China, Guatemala, India, Rwanda, and the sub-Saharan African countries. It shows that both rising sex ratios at birth and changing mortality patterns contributed to the observed crossover in fertility and represent a secular trend toward more masculine populations. In 1950, women began to outnumber men at around age 50 in all countries and areas, except in Guatemala and sub-Saharan Africa, where male survival was lower due to overall high mortality and the excess male mortality caused by war. The upward shift in the age at which women outnumber men is driven by higher sex ratios at birth (reflected in the increases of the purple bars), overall declines in mortality, and narrowing sex differences in mortality (both indicated by increasing green bars). While the sex ratio at birth naturally varies between 103 and 107 boys per 100 girls (25, 26), the Republic of Korea, China, and India show substantially higher sex ratios at birth. India is a special case, showing sustained excess numbers of men related to the continuously high sex ratio at birth and the narrow gender gap in mortality (due to comparatively high female mortality). Guatemala, Rwanda, and sub-Saharan Africa are experiencing sustained female skewed sex ratios at age 50 up to today.

While the overall trend is toward masculinization of reproductive-age populations, declining maternal mortality and conflict mortality work in the opposite direction. High maternal mortality historically created excess female deaths during reproductive years, contributing to more male-skewed sex ratios at these ages. As maternal mortality has declined globally (27), this source of excess female mortality has diminished, leading to more male-skewed sex ratios. The persistence of relatively high maternal mortality in sub-Saharan Africa contributes to the delayed or absent crossover to higher female TFR in this region. Moreover, Fig. 3 highlights the acute and lasting impact of conflict-related mortality in Guatemala (1980s) and Rwanda (early 1990s), where the mortality sex ratio bars exhibit sharp, transient dips reflecting elevated male mortality during periods of war and violence. For example, in the 1990s in Rwanda, a cohort exposed to wartime mortality would have exhibited a sex ratio of 85 men per 100 women at age 50, had mortality rates remained constant. These temporary shocks leave enduring imprints on population structure, skewing the age–sex composition for decades and affecting subsequent fertility dynamics.

## Discussion

This article has examined the difference in average reproduction between women and men in the past, the present, and the future around the world. While men used to have higher fertility than women, male-skewed population structures are expected to deflate reproduction numbers for men in the future, particularly in East Asia. Therefore, the majority of the world’s population will live in countries where the total fertility rate (TFR) for men is substantially lower than that for women from 2030 onward. A universal force leading to these sex imbalances in fertility is declining mortality, which sustains the male-skewed sex ratio at birth longer throughout life (28). A narrowing sex difference in mortality may also contribute to a masculinization of populations at reproductive ages. While this crossover seems to be universal, with the exception of sub-Saharan Africa, the excess men in the population will be rather modest in most countries. However, in some East Asian countries, where this crossover is reinforced by sex-selective abortion, the population can be substantially skewed.

We observe that male fertility historically exceeded female fertility, in line with previous findings (2, 3). With the onset of the fertility decline and as fertility reaches lower levels, the male and the female TFR cross, and female fertility starts exceeding male fertility. The timing of the crossover depends on the progress of the fertility transition. Crossovers occur first in Europe (1960s) and later in other contexts. In sub-Saharan Africa, some countries are not expected to experience a fertility crossover before 2100, highlighting the implications of recent fertility stalls (29, 30).

Male and female fertility can differ substantially, with the gap ranging from −60% to +130%. Extreme cases of a very high male TFR relative to female TFR (+100% or above) are mainly observed in the 1950s and 1960s in small populations like those in Lesotho, Tokelau, and Turks and Caicos Islands. Extreme cases of a very low male TFR relative to the female TFR are mostly observed in the 2020s and 2030s in countries like Qatar, the United Arab Emirates, the Maldives, and Oman.

The secular trend behind the crossover of the male and the female TFR is the masculinization of populations related to declining mortality levels, narrowing sex difference in mortality, and, in some countries, sex selective abortion leading to artificially high sex ratios at birth. The impact of mortality on the masculinization of populations has been observed for Western countries before by Schubert (2) and Dudel (28), and the issue of the missing women has been raised by Sen (31). Following Skirbekk and Spoorenberg (32), we acknowledge that mortality and fertility are fundamentally interconnected.

Beyond the secular trends driving the masculinization of populations, conflicts can have a strong and lasting impact on the sex ratios in populations, leading to a female surplus. Dependent on the intensity and duration of conflicts, population structures can be altered with potential implications for childbearing. Based on the period mortality rates for the Republic of Korea (1950) and Cambodia (1965), there would be 30 or 70 men per 100 women at age 30, respectively, if conflict mortality lasted in these countries for a cohort. The feminization of population structures may have positive effects on gender equality and the participation of women, but it also renders reproduction and partnering more difficult and selective for women.

The crossover marks the beginning of a new demographic reality, which will come with new opportunities and challenges. U.S.-based research shows some implications for marriage rates and fertility (9, 22). An excess number of men relative to women in reproductive ages will likely lead to higher levels of male childlessness and a steeper socioeconomic gradient of childlessness, as indicated by previous research (2, 5, 22, 33) and our supplementary analysis (*SI Appendix*, *TFR Ratios and Childlessness*). Increasing levels of male childlessness and excess numbers of men will have social and economic consequences, but those will likely only manifest in the extreme cases in the East Asian countries. Additional analysis showed that the average parental age gap widens if male-to-female TFR differences grow (*SI Appendix*, Fig. S14). Moreover, in East Asian countries, an excess of men in the population has been linked to increased rates of crime and sexually transmitted diseases (20, 21, 34).

Another finding of this study is that male fertility can be readily approximated with a regression-based approach using adult sex ratios and the female TFR. While Keilman et al. (23) suggested this approach and found only a lower model fit ($R^{2}=0.83$), we obtained excellent model performance statistics ($R^{2}=0.97$) and out-of-sample prediction error (RMSE=0.041), especially with a model accounting for age gaps between partners. We encourage future research to fine-tune this model.

### Limitations.

The study has two major limitations. First, male fertility is not directly observed, but is instead approximated through various indirect methods using population structures and female fertility rates. While these approximations yield a high out-of-sample fit (*Materials and methods*) and the observed data on male fertility have problems (35, 36), the models assume a certain relationship between the female TFR and the population structure compared to that of the male TFR, which may not hold. For instance, in special cases such as Qatar, where male-dominated labor migration leads to changing population structures, but not changing fertility, our results deviate from those reported in ref. 3. In these rare cases, our approximation is likely imperfect. Second, the main analysis only examines average fertility, e.g., total fertility rates, and in our supplementary analysis, we study age- and/or parity-specific fertility only for selective countries with readily available high-quality data. Previous research and supplementary analyses indicate that population imbalances mainly affect childlessness among the more abundant sex (2), but this may play out differently in other contexts.

### Outlook.

Our results suggest that sex differences in fertility are growing as a result of the masculinization of populations and that these shifts will come with challenges and opportunities. The challenges are mainly for men who remain childless, a status that is often associated with worse health and growing dependence on professional care in old age. We propose the following specific policy measures to address sex differences in fertility and their consequences (e.g., male childlessness, marriage market imbalances): strengthening the position of women in society to prevent sex-selective abortion; improving education and job creation to give childless and single men opportunities to pursue a career and to reduce their susceptibility to organized crime; and providing technical and institutional solutions for singles and childless individuals, such as friendship groups and legalizing of artificial reproductive technologies. Failing to address the needs of these men risk a cultural backlash against gender equality and societal conflicts.

## Materials and Methods

### Data.

We use data on age–sex specific population counts, annual birth counts by sex, and female TFRs from the United Nations World Population Prospects 2024 (WPP2024) (37), but male TFRs are not included in these data and are therefore estimated indirectly (3–5). WPP2024 provides comprehensive, internally consistent time series of population counts by single age and sex, births, deaths, and international migration for all countries and areas from 1950 to 2100. The dataset is freely accessible at https://population.un.org/wpp/. It integrates diverse data sources, including civil registration systems, sample registration, censuses, surveys, and national estimates, while explicitly accounting for biases such as undercoverage, underenumeration, and differential registration quality across age groups and regions (38). The population estimates are derived using the cohort component method, which reconstructs population dynamics through the population balancing equation. This approach ensures temporal consistency and enables reliable projections to 2100. Fertility and mortality indicators, including the $TFR_{w}$ and adult mortality, are generated via Bayesian hierarchical modeling that synthesizes heterogeneous data sources, adjusts for known measurement errors, and propagates uncertainty appropriately (39–41). This methodological framework enhances the reliability of estimates, particularly in data-sparse regions.

For male fertility, we use country- and time-specific TFR estimates derived from multiple sources: Schoen, Schubert, and Dudel (1, 4, 5) applied classical demographic methods to vital statistics, whereas Schoumaker (3) employed the own-child method using data from the Demographic and Health Surveys (DHS) (42). These estimates are harmonized to ensure comparability across countries and time periods.

In the main analysis, we use the medium scenario for the population, fertility, and mortality projections. In a robustness check, we exploited the different demographic scenarios estimated by the WPP2024 in order to understand the impact of assumptions regarding fertility, mortality, and migration on sex differences in fertility (*SI Appendix*, *Demographic Scenarios*). If fertility below age 18 would drop to zero across the world, the TFR ratios would be lower mainly in high fertility contexts like sub-Saharan Africa, Oceania, and Latin America, where teenage fertility is still substantial. If fertility was instantly set at replacement level, the TFR ratios in lower fertility countries would increase and the TFR ratios in higher fertility countries would drop, highlighting the impact of the female TFR on TFR ratios.

### Estimating Male Fertility.

We measure fertility using the total fertility rate for men ($TFR_{m}$) and for women ($TFR_{w}$). The total fertility rate is a period measure of fertility intensity, indicating the average number of children a woman or a man would have by the end of the reproductive period if she or he was subject to the age-specific fertility of a given year. The $TFR_{w}$ is obtained from the WPP2024, but the $TFR_{m}$ is not readily available or is subject to data deficiencies (35, 36), and therefore needs to be estimated.

The estimation of the $TFR_{m}$ follows Keilman et al. (23) and exploits a theoretical relationship of the $TFR_{m}$ to adult sex ratios and the $TFR_{w}$. The $TFR_{m}$ usually closely follows the $TFR_{w}$ (1), but unbalanced population structures can affect the reproduction of the more abundant sex (3, 5). Therefore, the $TFR_{m}$ is logarithmically related to the overall fertility level ($TFR_{w}$) and the sex difference in the size of the population at reproductive age (SR). The estimation is as follows:

[1] $$\begin{array}{c} \log\left(TFR_{m}\right)=\alpha +\beta_{1}\log\left(TFR_{w}\right)+\beta_{2}\log\left(SR\right)+\epsilon , \end{array}$$

where $\log(TFR_{m})$ is the logarithm of the TFR for men, $\log(TFR_{w})$ is the logarithm of the TFR for women, and log(SR) is the logarithm of the sex ratio at reproductive age.

We estimate three distinct models that differ in how they account for population sex ratios. The baseline model (Model 1) uses the sex ratio in the 20 to 39 age group, consistent with the approach previously employed by Keilman et al. (23). The postponement model (Model 2) adjusts for fertility postponement by estimating the sex ratio within the 25 to 44 age group (43, 44). The age gap model (Model 3) further refines this approach by accounting for the observed pattern of later childbearing among men: It calculates the sex ratio using men aged 25 to 44 and women aged 20 to 39, thereby capturing the age gap between partners at the time of childbirth (3, 45). While Models 1 and 2 compare the sex ratios within the same age groups, Model 3 introduces a temporal shift in the male age group to better reflect the demographic realities of partner age differences in contemporary fertility.

#### Model results.

All three models reach a better fit relative to the model in Keilman et al. (23) and the age gap model performs best. The baseline and postponement models yield a robust fit, as the $R^{2}$ are at 0.969 and 0.97, respectively, whereas the $R^{2}$ in Keilman et al. (23) was only 0.83. The age gap model performs best with an $R^{2}$ of 0.984. Furthermore, we perform out-of-sample validation using high-quality data from the Human Fertility Collection (1, 46) to evaluate the performance of the regression models and assess the problem of overfitting. Overall, the fit is good, reaching a root mean squared error (RMSE) of around 0.05. The best model fit is again found for Model 3 that accounts for the age gap, which has an RMSE = 0.041. The 90% prediction intervals are conservatively calibrated, as they include 98% of the $TFR_{m}$ observations.

The regression results are displayed in Table 1, indicating a positive correlation of the $TFR_{w}$ with the $TFR_{m}$ and a negative correlation of the adult sex ratio (SR) with the $TFR_{m}$ across models. The coefficients across the regression models in Table 1 are statistically significant, unlike the results in Keilman et al. (23), because of a larger sample size (*n*) and/or the better model fit ($R^{2}=0.983$). Hence, we use the complete regression equation for the approximation of the $TFR_{m}$. We now present the results for Model 3, which is the best performing model. If the population is balanced (sex ratio = 1) and the $TFR_{w}$ is at replacement level ($TFR_{w}=2.1$ births per woman), the $TFR_{m}$ is predicted to be slightly lower, at 2.09 births per man. However, if there are twice as many women as men in the reproductive age ranges (sex ratio = 0.5), the $TFR_{m}$ is predicted to increase to 3.31 (90% PI: 3.05 to 3.59). If there are half as many women as men in the reproductive age ranges (SR = 2), the $TFR_{m}$ drops to 1.32 (90% PI: 1.22 to 1.44), holding the $TFR_{w}$ at replacement level. Holding the population balanced, the impact of the $TFR_{w}$ is negative, which implies that at a lower $TFR_{w}$ of 1.0, the $TFR_{m}$ equals 0.92 (90% PI: 0.85 to 1.00), and if the $TFR_{w}$ increases to 3.0, the $TFR_{m}$ reaches 3.1 (90% PI: 2.86 to 3.36).

**Table 1.** Regression table presenting the results from the regression in Eq. **1**

	Dependent variable: log TFR men		
	(1)	(2)	(3)
	Baseline	Postponement	Age gap
log TFR women	1.182^∗∗∗^ (1.175, 1.190)	1.197^∗∗∗^ (1.190, 1.205)	1.101^∗∗∗^ (1.095, 1.107)
log SR (20-39)	−0.887^∗∗∗^ (−0.922, −0.852)		
log SR (25-44)		−0.849^∗∗∗^ (−0.884, −0.814)	
log $\frac{{\text{men}}_{25-44}}{{\text{women}}_{20-39}}$			−0.661^∗∗∗^ (−0.675, −0.646)
Intercept	−0.092^∗∗∗^ (−0.098, −0.086)	−0.114^∗∗∗^ (−0.119, −0.108)	−0.078^∗∗∗^ (−0.082, −0.074)
Observations	4,024	4,024	4,024
R^2^	0.968	0.968	0.983
Adjusted R^2^	0.968	0.968	0.983

The predictor variables are the total fertility rate for women (logarithm) and the sex ratio at ages 20 to 39 (logarithm). The outcome variable is the total fertility rate for men (logarithm). The *Top* panel presents the regression coefficients and the *Bottom* panel presents the model metrics. *Note:*^∗^*P* < 0.1; ^∗∗^*P* < 0.05; and ^∗∗∗^*P* < 0.01.

In a robustness check, we accounted for fundamental uncertainty in the regression model and used 90% prediction intervals, which blurred the picture a bit (*SI Appendix*, *Prediction Intervals*). The TFR differences for the Caribbean, Central America, and the less developed regions became indistinguishable from zero due to prediction uncertainty, but the findings for high-income countries and sub-Sahara African and East Asian countries remained robust.

### Standardization.

Beyond the regression-based approach, we employ demographic standardization to isolate the impact of sex specific population structures on observed sex differences in total fertility rates (TFRs). Standardization is a widely used technique to disentangle the influence of population composition, such as age and sex structure, on aggregate demographic indicators (47). Here, we apply the distribution of births by maternal age to the male population structure, effectively estimating what the male TFR would be if men experienced the same fertility schedule as that of women, but were exposed to the actual age distribution of the male population. The standardized TFR is computed as

[2] $$\begin{array}{c} {\text{TFR}}_{\mathrm{std}}=\sum_{x=15}^{55}\frac{B_{x}}{P_{x}^{m}}, \end{array}$$

where $B_{x}$ denotes the number of births to mothers aged x, and $P_{x}^{m}$ is the male population aged x in the reproductive age range (15 to 55 y). This approach implicitly assumes that the fertility schedule is identical across sexes—a simplification that does not hold in reality. While empirical evidence shows that male fertility schedules are typically shifted to older ages, exhibit a broader reproductive window, and decline more gradually after the peak compared to female schedules (3, 6), the standardization reveals how much of the observed difference in the TFRs of men and women is solely attributable to the skew in sex ratios within age groups at reproductive ages. For selected countries, we also conducted further analyses accounting for the age differences between men and women, based on data from Dudel and Klüsener (1). The findings match the regression results very closely (*SI Appendix*, *Age Gap Approach*).

### Untangling the Demographic Drivers of Sex Imbalances.

To disentangle the contributions of sex ratios at birth and sex-specific mortality to changing population structures, we leverage sex-specific life tables and sex ratios at birth from the WPP2024. We construct age-specific sex ratios by applying a synthetic cohort approach: starting from the sex ratio at birth (e.g., the number of male births per 100 female births), we project the survival of males and females through each age group using the corresponding sex-specific life tables (the probability of surviving to the next age, p(x)). Specifically, we set the radix for males to the observed sex ratio at birth (e.g., 105 males per 100 females) while setting the radix for females to 100, and then we apply the cumulative product of age-specific survival probabilities,

[3] $$\begin{array}{c} SR_{t}\left(x\right)=100\times \underset{\text{Birth contribution}}{\underbrace{\left(\frac{B_{m,t}}{B_{w,t}}\right)}}\times \underset{\text{Mortality contribution}}{\underbrace{\left(\frac{\prod_{i=0}^{x}p_{m,t}\left(x\right)}{\prod_{i=0}^{x}p_{w,t}\left(x\right)}\right)}}, \end{array}$$

where $B_{m}$ and $B_{w}$ are the numbers of male and female births, respectively, and p(x) is the age-specific probability of surviving to the next age. This approach allows us to compute the age-specific sex ratio—i.e., the number of men per 100 women—at each age, reflecting the cumulative impact of imbalanced sex ratios at birth and sex-specific mortality across the life course. By using this approach, we effectively isolate the demographic forces shaping the sex composition of the reproductive age population, neutralizing the influence of international migration, which is not directly modeled in this decomposition.

## Supplementary Material

Appendix 01 (PDF)

## Data Availability

All code and data required to replicate the main and supplementary results of the article can be found here: https://github.com/Henrik-Alexander/global_birth_squeezes (48). All other data are included in the manuscript and/or *SI Appendix*.
