# Supplementary material for: Masculinization of populations reverses sex differences in fertility
Source: Proc Natl Acad Sci U S A. 2026 Apr 20;123(17):e2533317123. doi: 10.1073/pnas.2533317123 (PMC13123817; doi:10.1073/pnas.2533317123)
Supplement: Supplementary file 1 — Appendix 01 (PDF) [file pnas.2533317123.sapp.pdf]

Supporting Information for

Masculinization of populations reverses sex difference in fertility

Henrik-Alexander Schubert, Thomas Spoorenberg, Christian Dudel and Vegard Fykse Skirbekk

Corresponding Author: Henrik-Alexander Schubert.
E-mail: schubert@demogr.mpg.de

This PDF file includes:

Supporting text
Figs. S1 to S19
Tables S1 to S6
SI References

Supporting Information Text

1. Regression-based approach

Male fertility is a topic in demography that is widely sidelined for some substantial and methodological reasons, despite prominent calls to bring men back in (1). Among the reasons are data quality, data availability, and the utility male fertility estimates, given that most population projections take a female-centered approach. The quality of data on male fertility is usually inferior to that of data on female fertility because men tend to under-report their number of children when they do not live with them (2). Moreover, vital statistics data usually contain a large fraction of missing values on paternal age, or paternal age is only reported fo married couples (3). Recent methodological advancements have attempted to overcome these problems (3, 4). This has resulted in a wider availability of data on male fertility (5–7), providing new insights.

We obtain male fertility data through an indirect demographic approach proposed by Keilman et al. (8), which relates the TFR for men to the TFR for women and the sex ratio at reproductive ages, see Equation 1. We have adjusted the model so that it is better suited for contemporary estimations. The first adjustment (Model 2) accounts for fertility postponement, shifting the age window for estimating the reproductive sex ratio from 20–39 to 25–44, which better captures the fertility intensities after global fertility postponement (9). The second alternative (Model 3) also accounts for the age gaps between fathers and mothers by estimating the sex ratio between 25-44-year-old men and 20-39-year-old women (10).

The model is trained on male fertility data described in Table S1 and follows equation 1. The data sources contain individual-level birth records with information on paternal age and maternal age (see top panel of Table S1), or readily available data at the country level (see bottom panel of Table S1). If the paternal age was missing, the information was imputed using the conditional approach proposed in Dudel and Klüsener (3). More information on the data curation of the subnational data can be found in Schubert and Dudel (5); information on the data from the Human Fertility Collection can be obtained from Dudel and Klüsener (6); and the estimation method for the DHS data is described in Schoumaker (4). The data from the Human Fertility Collection, which arguably represent the gold standard of male fertility data, have been excluded from the model training and are only used for the model validation, see Section 2. The model appears as follows,

$$log(TFR_m) = \alpha + \beta_1 log(TFR_w) + \beta_2 log(SR) + \epsilon \quad [1]$$

where $log(TFR_m)$ is the logarithm of the TFR for men, $log(TFR_w)$ is the logarithm of the TFR for women, and $log(SR)$ is the logarithm of the sex ratio at reproductive age.

The use of an indirect demographic approach relies on several assumptions. First, the approach assumes that the model captures a large fraction of variation in the male TFR. This is confirmed by a high R^2. Second, it assumes that the relationship is the same in the training data as in the prediction data. The training data contain almost all countries and areas around the world and several time episodes, which makes them a good representation of different demographic contexts. The out-of-sample prediction confirms that the model is able to predict the TFR for men outside the sample, see Section 2.

2. Out-of-sample validation

The model from the indirect approach to estimating the TFR_m was evaluated using out-of-sample validation on data from the Human Fertility Collection (6), which were withheld from the model training. The predicted values and the 90% prediction intervals are obtained from regression models using data from the WPP2024 on the adult sex ratios and the TFR_w. We are thus able to compare the observed to the predicted value, and to test the calibration of the uncertainty, see Figure S1. The figure shows the observed TFR for men on the x-axis and the predicted TFR for men on the y-axis. Large deviations from the diagonal line indicate a larger prediction error. The vertical bars indicate the 90% prediction intervals. Model 3, in the bottom panel labeled *age gap model*, has the best prediction performance (smallest gaps between the predicted and the observed value) and the best calibrated prediction intervals. Furthermore, Figure S2 reveals that the average prediction error is the smallest.

3. Prediction uncertainty

We perform a robustness check accounting for the prediction uncertainty in the regression model to ensure the robustness of the main results, in particular of the declining trend in the TFR_m relative to the TFR_w and the fertility crossovers, and to understand the uncertainty in the regression model. The prediction interval very likely (with a probability of $1 - \alpha$) contains the random future observation y_0 (11). We set $\alpha = 0.05$ and the prediction intervals are estimated in the following way:

$$\boldsymbol{x}_0'\hat{\boldsymbol{\beta}} \pm t_{n-p}(1 - \alpha/2)\hat{\sigma}(1 + x_0'(\boldsymbol{X'X})^{-1}\boldsymbol{x_0})^{1/2} \quad [2]$$

where $\boldsymbol{x}_0'$ is the value for the predictor variables, $\hat{\boldsymbol{\beta}}$ is a vector of the regression coefficients from model 1, t_{n-p} is the t-value from the regression coefficients, and $\hat{\sigma}$ is the standard deviation for the regression coefficients. Figures S3 and S4 show the uncertainty around the predicted values of the TFR of men, largely confirming our main results.

4. Demographic scenarios

Furthermore, we account for different demographic scenarios of the future developments of population structures and fertility rates implemented in the WPP2024 (for details on the estimation, see 12). We include the following scenarios:

- Accelerated decline of the adolescent birth rate (ABR) with recovery: Accelerated ABR decline with recovery of half of reduced fertility once cohorts have aged 10 years
- Accelerated ABR decline: Age-specific fertility below age 20 declines by 20% per year until the ABR is below 10 births per 1,000 women aged 15-19
- Constant mortality
- Fertility scenarios: Low fertility, high fertility, instant replacement fertility, constant fertility, no fertility below age 18
- Instant replacement zero migration
- 80% prediction intervals: Lower 80 PI, upper 80 PI
- 95% prediction intervals: Lower 95 PI, upper 95 PI
- Medium: The mean probabilistic projections for fertility and mortality, and the median probabilistic projections for net migration
- Momentum: Instant replacement fertility as of 2024, constant mortality as of 2024, zero migration from 2024
- Freeze rate: This approach assumes no change in the demographic parameters
- Zero migration

The results, displayed in Figures S5, and S6, show that the trends are largely parallel, but the scenarios differ in terms of the magnitude of the difference between the TFR for men and the TFR for women. Scenarios with slower fertility decline and higher population growth rates, such as the high scenario and the upper uncertainty boundaries, show more muted declines of the TFR for men relative to the TFR for women. The instant replacement fertility scenario differs in the impact dependent on the current fertility level. If instant replacement fertility leads to a sudden increase in fertility, which is the case for low fertility countries, it would also lead to higher TFR_m relative to the TFR_w, see high-income panel in Figure S5. However, in regions with above replacement fertility, for instance in sub-Saharan Africa, the relative difference declines more sharply, see bottom left panel in Figure S5. The low population growth scenarios and the lower prediction intervals generally show a lower TFR_m relative to the TFR_w.

To illustrate the impact of a WPP2024 scenario relative to the medium scenario used in the main results, we estimate the absolute difference between the scenario at hand and the medium-scenario in Figures S7 and S8. Thus, values higher than zero indicate that this specific scenario may lead to a larger relative difference than in the medium scenario. We can further distinguish between statistical scenarios (incorporating projection uncertainty) and demographic scenarios (modeling different deterministic scenarios for the evolution of demographic behavior), see Figures S9 and S10.

5. Standardization

We use standardization as a tool to demonstrate the impact of population sex differences in the same age-groups on the difference in the total fertility rate. The interpretation is as follows: the estimated TFR for men would be the result if the mother and the father were always exactly the same age. While this assumption is not realistic (for evidence on parental age gaps, see 10, and Section 7), it illustrates the impact of sex differences in the population structures. The results presented in Figure S11 corroborate the results from the indirect estimation model showing similar trends towards a lower TFR_m relative to the TFR_w over time, but the differences are a bit more muted, because those patterns are more reinforced by the actual age differences between parents.

6. Age gap approach

The standardization simplifies the estimation by assuming that the mother is exactly the same age as the father, but one could also use empirical data on the age differences between the parents to obtain a more realistic estimation of the TFR_m that relaxes the assumption regarding the age differences. We do this in the age gap approach by drawing on data on the conditional distribution of births by father's age y for each maternal age group x from Dudel and Klüsener (6), and present the results in Figure S12. The conditional distribution $P(y \mid x)$ can be defined as the probability that a birth to a mother aged x is happening to a father aged y. The sum of the conditional distribution for each maternal age is 1: $1 = \sum_{i=15}^{55} P(y \mid x)$. Using such conditional distributions, we distribute the births using the following estimation: $B(y) = \sum_{i=15}^{55} B(x) \cdot P(y \mid x)$. The standardization approach is a special case of the age gap approach in which for the case $x = y \Rightarrow P(y \mid x) = 1$ and for all other ages y is $P(y \mid x) = 0$, so that births are not shifted to other age groups.

The results from the standardization, age gap, and regression-based approaches show similar trends overall, see Figure S12. The results from the age gap approach and the regression-based approach are particularly close, which is that our regression-based approach, which is used for the main results, captures well both sex imbalances and age gaps. Moreover, the 90% prediction intervals contain the results from all three approaches, which indicates that the model uncertainty is well calibrated. The TFR ratio from the standardization approach is usually slightly lower than that from the other approaches, which shows that age gaps partially offset the impact of population imbalances in age groups.

7. TFR ratios and fertility timing

We investigate the role of age differences between men and women for the evolution of population sex ratios, see Figure S13. The results indicate that the age gap might have been a powerful counterforce to population sex ratios in the past, but its impact is weakening, as fertility levels are predicted to decline globally, which will likely result in smaller age gaps between partners. The simple sex ratio is estimated by dividing the number of men aged 16 to 49 by the number of women in the same age group ($\frac{\sum_{y=15}^{49} P_m(y)}{\sum_{x=15}^{49} P_f(x)}$). In the age gap population sex ratio, we use the same population counts for women, but for men, the age window is shifted by a factor δ, which is the average parental age gap ($\frac{\sum_{y=15+\delta}^{49+\delta} P_m(y)}{\sum_{x=15}^{49} P_f(x)}$). The average parental age gap is obtained using predictions from a polynomial regression model that regresses the average age gap between parents on the TFR for women with a linear and polynomial term, see equation 3. This model is then used to predict the average age gap in all countries based on the TFR rates from the WPP2024.

$$(MAF - MAC) = \beta_0 + \beta_1 TFR_W + \beta_2 TFR_w^2 + \epsilon \quad [3]$$

Moreover, we study the potential adjustment of fertility timing as a consequence of population imbalances using all available data on the mean age at childbearing/fatherhood from Schoumaker (7), Dudel and Klüsener (6), Max Planck Institute for Demographic Research and Vienna Institute of Demography (13), and Schubert and Dudel (5) and our estimated TFR ratios, see Figure S14. The results indicate a relationship, with the smallest average age gap being observed in countries with balanced population structures, and growing average parental age gaps with both male-skewed and female-skewed populations.

The results should be interpreted with caution, however, because of the methodological challenges associated with it. For instance, the relationship may go both ways, because larger average parental age differences *en pair* with population growth will lead to larger TFR ratios, indicating a reversed relationship (7). This is because in a growing population, the exposures are larger in younger age groups relative to older age groups, so that the TFR for men is more inflated as most births will be related to small exposures, while for women the mass of births is related to larger exposures. This observation led to the stable population approximation by Schoumaker (7),

$$\frac{TFR_m}{TFR_f} \approx \frac{1}{SRB} \cdot \frac{p(MAC)}{p(MAF)} \cdot exp(r \cdot (T^m - T^f)), \quad [4]$$

where SRB is the sex ratio at birth, $p(MAC)$ and $p(MAF)$ are the probability of surviving until the mean age at childbearing, r is the growth rate, and T is the generation length. Here, the terms $p(MAC)$ and $p(MAF)$, as well as the generation length T capture the impact of the average parental age gap, which is multiplicatively related to the population growth rate. Moreover, the relationship between age at childbearing and TFR ratios is confounded by the overall fertility level as well as traditional attitudes.

8. TFR ratios and childlessness

We have collected readily-available country-level data on childlessness for men and women from Tanturri et al. (14) for 20 countries in 2010, and relate those numbers to the estimated female-to-male TFR ratios ($\frac{TFR_m}{TFR_w}$), see Figure S18. The data consists of estimates on ultimate childlessness (women aged 45-50 and men aged 50-55) based on the Gender and Generation Survey, a demographic and social survey conducted in Europe, and administrative register data. The challenge is that the TFR ratios are period measures and childlessness is a cohort outcome. To address this we employ an approximation: by inserting a 15-year lag, we relate the average TFR ratios from the 1990s to the childlessness ratio in 2010 in order, to approximate the population exposure over the reproductive period of this cohort. We use a 15-year difference because the mass of the birth distribution is around age 30 and ultimate childlessness refers to ages 45-55, i.e. approximately 15 years later. The figure confirms the results from Schubert and Dudel (15) indicating a relationship between unbalanced population structures and amplified male childlessness. We acknowledge the uncertainty related to this approach. The results suggest that the higher the TFR ratio, i.e. the more female skewed the population, the lower the level of male childlessness relative to female childlessness. Furthermore, in Schubert and Dudel (5), the authors relate the regional population sex ratio at age 30 to the probability of childlessness at age 45 in Finland, supporting a relationship between male-skewed population structures and increases in male childlessness.

9. Decomposition of drivers of TFR ratio change

The change in the TFR ratio can be related to both changes in population structures and/or the fertility level. To clarify the relative contributions of fertility levels versus sex ratios by age, we estimated a counterfactual simulation that holds each factor constant, in turn, at 2025 levels, see Figure S19. The observed TFR ratio is estimated the following way:

$$\left(\frac{TFR_m}{TFR_w}\right)_{observed} = \frac{exp(\alpha) \cdot \beta_1 TFR_{observed} \cdot \beta_2 SR_{observed}}{TFR^w_{observed}}, \quad [5]$$

where the TFR ratio is related to the parameters from the regression and the observed values of the TFR and the SR. Using the equation and replacing the population sex ratio with the value from 2025 will provide a value for the counterfactual scenario in which the population distribution remained at the current value from 2025. The scenario is estimated the following way,

$$\left(\frac{TFR_m}{TFR_w}\right)_{\text{population from 2025}} = \frac{exp(\alpha) \cdot \beta_1 TFR_{observed} \cdot \beta_2 SR_{\text{Population from 2025}}}{TFR^w_{observed}}, \quad [6]$$

where $SR_{\text{Population from 2025}}$ is the counterfactual value for the population sex ratio from the year 2025.

The impact of the evolution of the TFR can be studied analogously. Simply exchanging the observed TFR with the TFR value from the year 2025 in the nominator and the denominator, as done below in

$$\left(\frac{TFR_m}{TFR_w}\right)_{\text{TFR from 2025}} = \frac{exp(\alpha) \cdot \beta_1 TFR_{\text{TFR from 2025}} \cdot \beta_2 SR_{observed}}{TFR^w_{\text{TFR from 2025}}}. \quad [7]$$

The results in Figure S19 indicate that the evolution of both the TFR for women and population sex ratios have contributed to the development of the TFR ratio. The interpretation is simply that the larger the difference is between the observed value (solid line) and the counterfactual scenario (dotted line), the more impact the change in this component has. In countries with highly skewed population structures due to sex-selective abortion, e.g., India and China, the blue dotted line is usually further away from the solid line, indicating that the population sex ratio has been a stronger driver of changes in the TFR ratio than the female TFR. For example in India (top row left column panel) to the left of the vertical dashed line (before 2025), the counterfactual lines are below the observed line, indicating that both the population sex ratio and the female TFR were higher in the past, exerting a stronger upward pressure on the TFR ratio than in 2025. In 2000, changing SRs contributed 4.22% and changing TFRs contributed 5.29% to the lower TFR ratio compared to 2025. To the right of the vertical dashed line (after 2025), the dotted blue line is above the solid line, indicating that the population sex ratio will have a stronger negative impact on the TFR ratio than in 2025. The TFR ratio would be 5.25% higher in 2040 if the population SR were to stay at the same level as in 2025.

Materials

Table S1. The table summarizes the male fertility data used in the study to train the regression models, providing information on the country, the observation period, the spatial unit, the number of spatial units, and a link to the source.

Country	Period	Level	Units	Source
Australia	1990-2020	States, Territories	8	explore.data.abs.gov.au
Colombia	1998-2020	Departments	32	https://microdatos.dane.gov.co
Finland	1990-2020	Regions	19	https://www.stat.fi/
France	1989-2013	Regions	13	insee.fr/fr/statistiques
Germany	1990-2018	States	16	https://www.destatis.de
Mexico [a]	1990-2021	States	32	inegi.org.mx/programas/natalidad
USA	1969-2004	States	51	https://data.nber.org/natality/
Spain	1998-2020	Provinces	32	https://www.ine.es/
Human Fertility Collection	1968-2016	Countries	17	https://www.fertilitydata.org/Data/DataAvailability#MTOTTable
Schoumaker's fertility data	2010	Countries	163	https://perso.uclouvain.be/bruno.schoumaker/data/
Schoen's fertility data	1963-1974	Countries	22	https://www.sciencedirect.com/science/article/pii/0049089X85900043

[a] The time-series is not complete for all states. There is no information for Aguascalientes, Baja California, Baja California Sur, Campeche, Chiapas, Chihuahua, Ciudad de México, Coahuila de Zaragoza and Colima after 2015.

Table S2. This table summarizes the TFR for wommen from the WPP2024 over the entire time period by decade. The first column provides the variable name, the second column indicates the decade, the third to seventh columns provide the decade-mean, standard deviation, minimum, and maximum.

Indicator	Decade	μ	SD	Minimum	Maximum
TFRw	1950-1960	5.352	1.621	1.836	8.253
TFRw	1960-1970	5.237	1.736	1.632	8.249
TFRw	1970-1980	4.566	1.915	1.337	8.597
TFRw	1980-1990	3.976	1.885	1.133	8.770
TFRw	1990-2000	3.357	1.702	0.932	8.377
TFRw	2000-2010	2.911	1.506	0.798	7.700
TFRw	2010-2020	2.638	1.295	0.705	7.531
TFRw	2020-2030	2.299	1.085	0.661	6.325
TFRw	2030-2040	2.108	0.830	0.753	5.339
TFRw	2040-2050	1.981	0.632	0.839	4.461
TFRw	2050-2060	1.895	0.495	0.914	3.780
TFRw	2060-2070	1.834	0.395	0.976	3.268
TFRw	2070-2080	1.789	0.321	1.034	2.882
TFRw	2080-2090	1.756	0.265	1.085	2.612
TFRw	2090-2100	1.731	0.221	1.125	2.403

Table S3. This table summarizes the data from the WPP2024 and the estimated TFR for men over the entire time period. The first column provides the variable name, the second column indicates the decade, the third to seventh columns provide the decade-mean, standard deviation, minimum, and maximum.

Indicator	Decade	μ	SD	Minimum	Maximum
TFRm	1950-1960	6.499	2.224	1.885	11.620
TFRm	1960-1970	6.306	2.405	1.696	11.317
TFRm	1970-1980	5.525	2.654	1.061	11.137
TFRm	1980-1990	4.812	2.682	1.138	11.683
TFRm	1990-2000	3.912	2.425	0.909	11.322
TFRm	2000-2010	3.299	2.124	0.759	9.931
TFRm	2010-2020	2.905	1.805	0.560	9.351
TFRm	2020-2030	2.446	1.501	0.574	9.520
TFRm	2030-2040	2.189	1.136	0.659	7.246
TFRm	2040-2050	1.981	0.840	0.726	5.279
TFRm	2050-2060	1.830	0.648	0.744	4.409
TFRm	2060-2070	1.770	0.505	0.759	3.648
TFRm	2070-2080	1.708	0.409	0.839	3.103
TFRm	2080-2090	1.652	0.341	0.894	2.761
TFRm	2090-2100	1.627	0.283	0.894	2.478

Table S4. This table summarizes the data from the WPP2024 and the estimated adult sex ratio (men aged 24-45 to women aged 20-39) for the entire time period. The first column provides the variable name, the second column indicates the decade, the third to sixth columns provide the decade-mean, standard deviation, minimum, and maximum.

Indicator	Decade	μ	SD	Minimum	Maximum
Adult Sex Ratio	1950-1960	-0.139	0.160	-0.838	0.893
Adult Sex Ratio	1960-1970	-0.119	0.161	-0.713	1.361
Adult Sex Ratio	1970-1980	-0.134	0.173	-0.750	1.380
Adult Sex Ratio	1980-1990	-0.139	0.172	-0.670	1.346
Adult Sex Ratio	1990-2000	-0.090	0.167	-0.505	1.097
Adult Sex Ratio	2000-2010	-0.065	0.174	-0.619	1.456
Adult Sex Ratio	2010-2020	-0.039	0.187	-0.490	1.383
Adult Sex Ratio	2020-2030	-0.004	0.192	-0.556	1.324
Adult Sex Ratio	2030-2040	0.001	0.169	-0.539	1.141
Adult Sex Ratio	2040-2050	0.031	0.140	-0.304	0.960
Adult Sex Ratio	2050-2060	0.065	0.128	-0.180	0.915
Adult Sex Ratio	2060-2070	0.049	0.109	-0.162	0.797
Adult Sex Ratio	2070-2080	0.056	0.100	-0.165	0.706
Adult Sex Ratio	2080-2090	0.073	0.094	-0.109	0.640
Adult Sex Ratio	2090-2100	0.068	0.085	-0.098	0.596

Table S5. This table summarizes the difference between the TFR_m and the TFR_w in the period between 1950 and 2100 by decade. The first column provides the variable name, the second column indicates the decade, the third to sixth columns provide the decade-mean, standard deviation, minimum, and maximum.

Indicator	Decade	μ	SD	Minimum	Maximum
TFR Difference	1950-1960	0.201	0.132	-0.387	0.921
TFR Difference	1960-1970	0.183	0.133	-0.560	0.716
TFR Difference	1970-1980	0.176	0.144	-0.553	0.753
TFR Difference	1980-1990	0.162	0.151	-0.546	0.731
TFR Difference	1990-2000	0.106	0.149	-0.498	0.492
TFR Difference	2000-2010	0.073	0.148	-0.616	0.586
TFR Difference	2010-2020	0.047	0.146	-0.605	0.520
TFR Difference	2020-2030	0.010	0.147	-0.593	0.599
TFR Difference	2030-2040	-0.001	0.129	-0.542	0.469
TFR Difference	2040-2050	-0.028	0.104	-0.485	0.261
TFR Difference	2050-2060	-0.054	0.091	-0.469	0.185
TFR Difference	2060-2070	-0.047	0.075	-0.427	0.127
TFR Difference	2070-2080	-0.054	0.067	-0.391	0.094
TFR Difference	2080-2090	-0.066	0.062	-0.364	0.072
TFR Difference	2090-2100	-0.064	0.055	-0.341	0.047

Table S6. This table summarizes the frequency of crossovers over the entire time period. The first column provides the variable name, the second column indicates the decade, the third to sixth columns provide the decade-mean, standard deviation, minimum, and maximum.

Indicator	Decade	μ	SD	Minimum	Maximum
Crossover	1950-1960	0.004	0.062	0	1
Crossover	1960-1970	0.010	0.100	0	1
Crossover	1970-1980	0.007	0.084	0	1
Crossover	1980-1990	0.016	0.127	0	1
Crossover	1990-2000	0.017	0.128	0	1
Crossover	2000-2010	0.005	0.071	0	1
Crossover	2010-2020	0.014	0.119	0	1
Crossover	2020-2030	0.011	0.103	0	1
Crossover	2030-2040	0.007	0.082	0	1
Crossover	2040-2050	0.013	0.111	0	1
Crossover	2050-2060	0.008	0.088	0	1
Crossover	2060-2070	0.005	0.071	0	1
Crossover	2070-2080	0.005	0.071	0	1
Crossover	2080-2090	0.005	0.068	0	1
Crossover	2090-2100	0.004	0.065	0	1

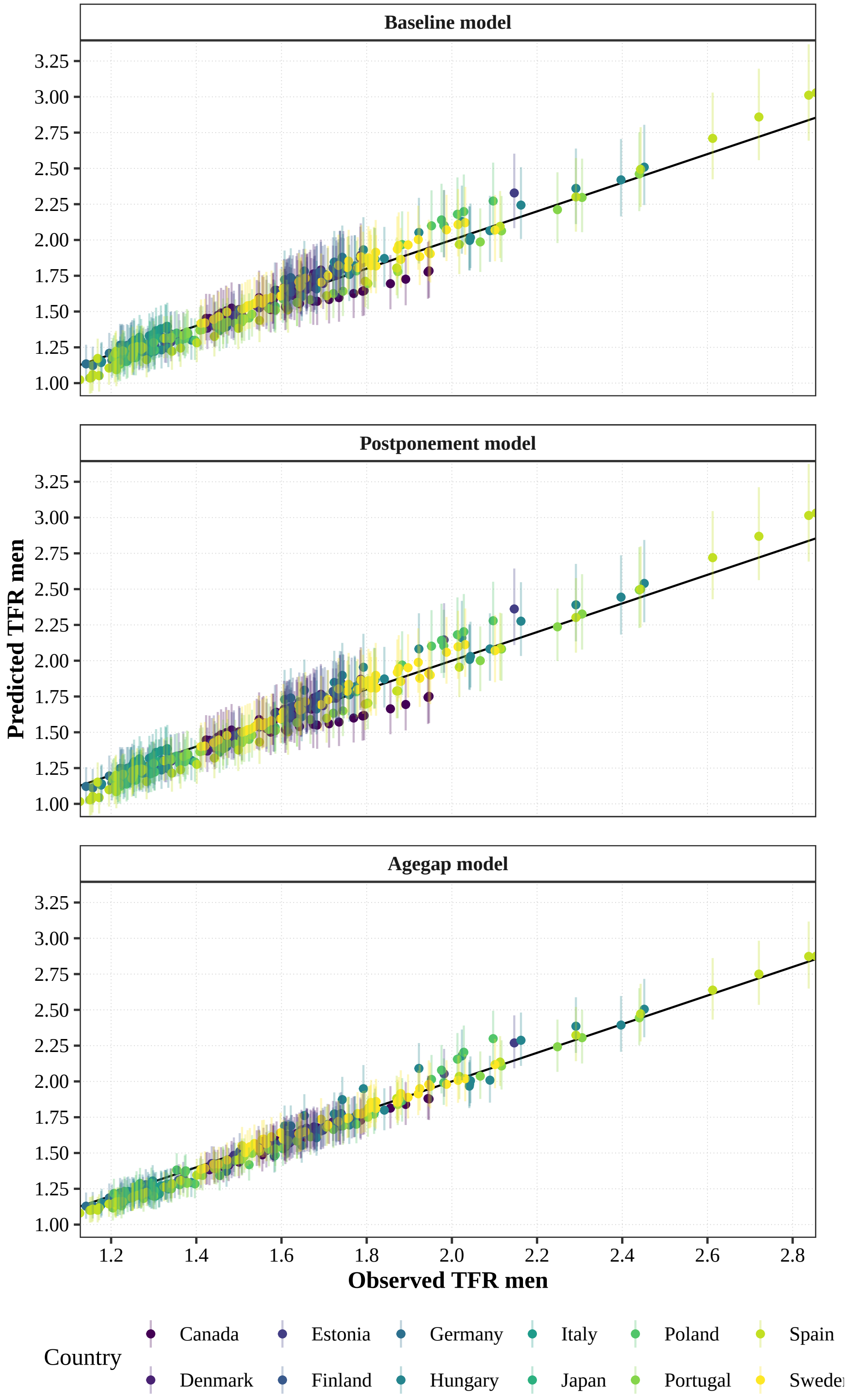

Fig. S1. This figure illustrates the out-of-sample validation of the baseline model, the postponement model and the age-gap model on data from the Human Fertility Collection (6). The x-axis shows the observed TFR_m from the Human Fertility Collection, while the y-axis shows the predicted TFR_m and the 90%-prediction intervals from the different regression models using data on adult sex ratios and TFR_w from the WPP2024.

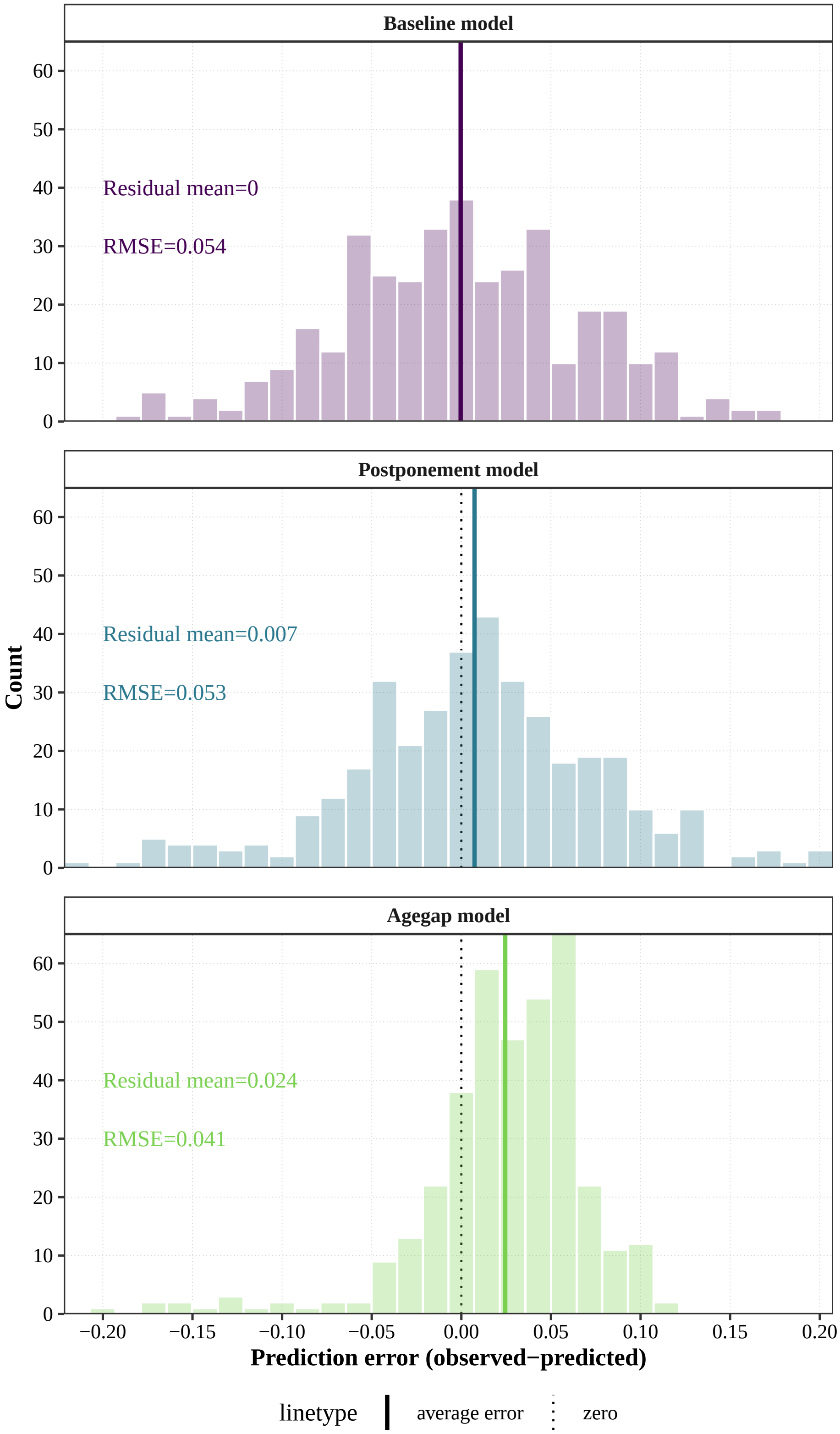

Fig. S2. Out-of sample validation error of the regression-based approach using data from WPP2024 on the data in Dudel and Klüsener (6). The x-axis provides e_i error between the observed and predicted value, and the y-axis shows the count of observations.

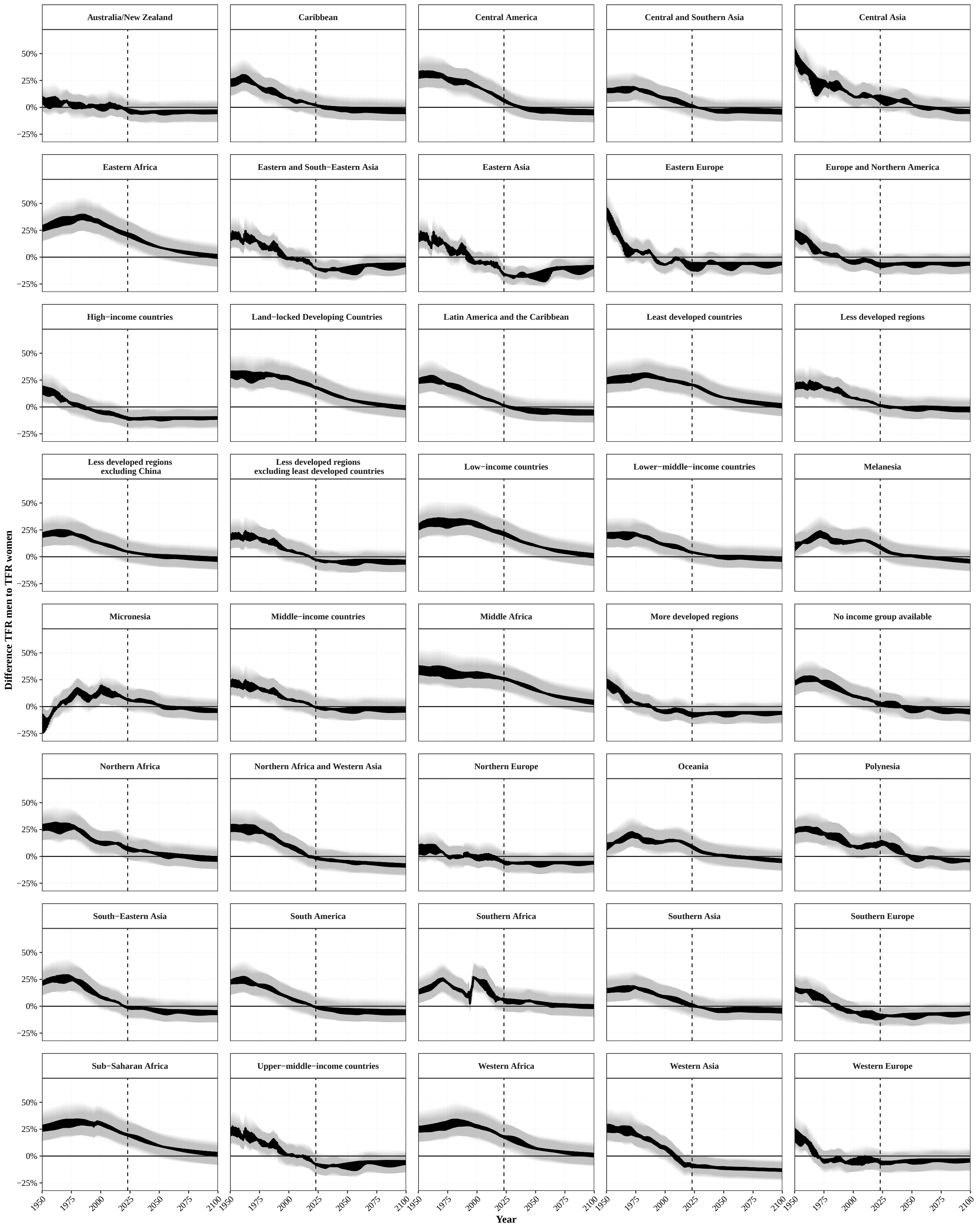

Fig. S3. This figure illustrates the relative difference between the TFRm to TFRw (y-axis) in the period between 1950 and 2100 (x-axis) accounting for the prediction uncertainty (grey shading) for the different regional classifications.

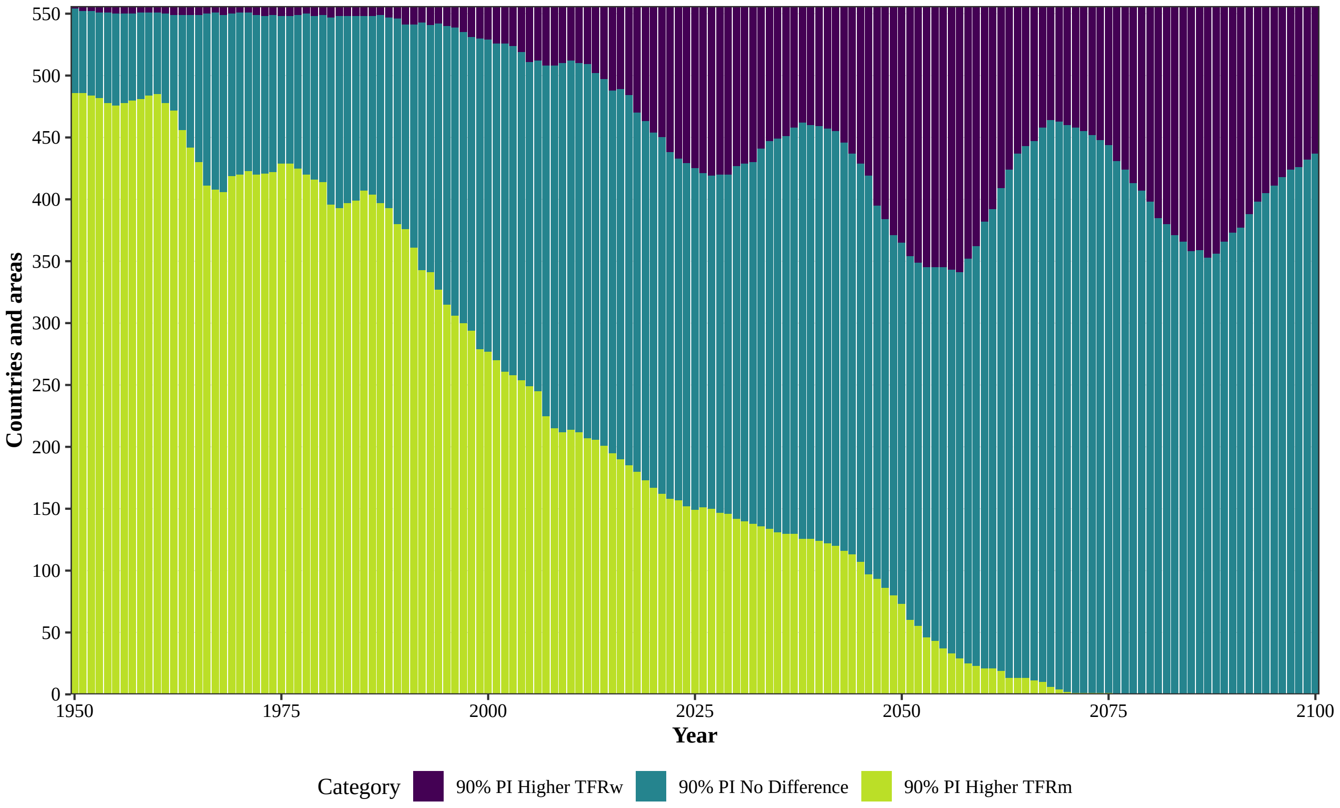

Fig. S4. This figure illustrates on the y-axis the number of countries and areas with higher TFRm than TFRw (green), higher TFRw than TFRm (purple) and countries and areas where the difference is not statistically significant (blue) in the period between 1950 and 2100 (x-axis) accounting for the prediction uncertainty.

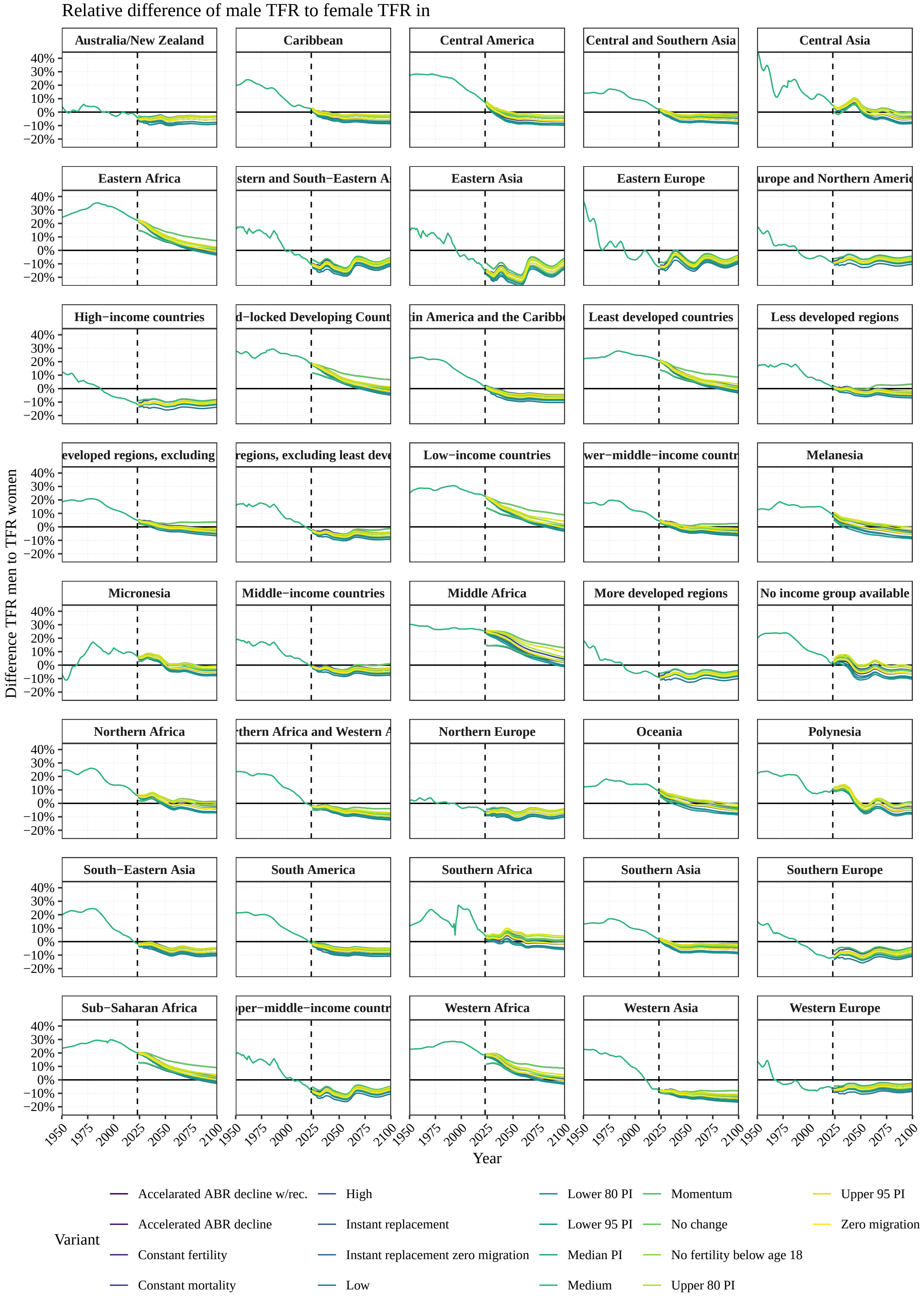

Fig. S5. This figure illustrates the impact of different WPP2024-scenarios (colours) on the relative difference between the TFR_m to the TFR_w in per cent (y-axis) across country groups (different panels) between 1950 and 2100 (x-axis). Values higher than 0 indicate higher TFR_m relative to the TFR_w, and values below 1 indicate higher TFR_w relative to the TFR_m.

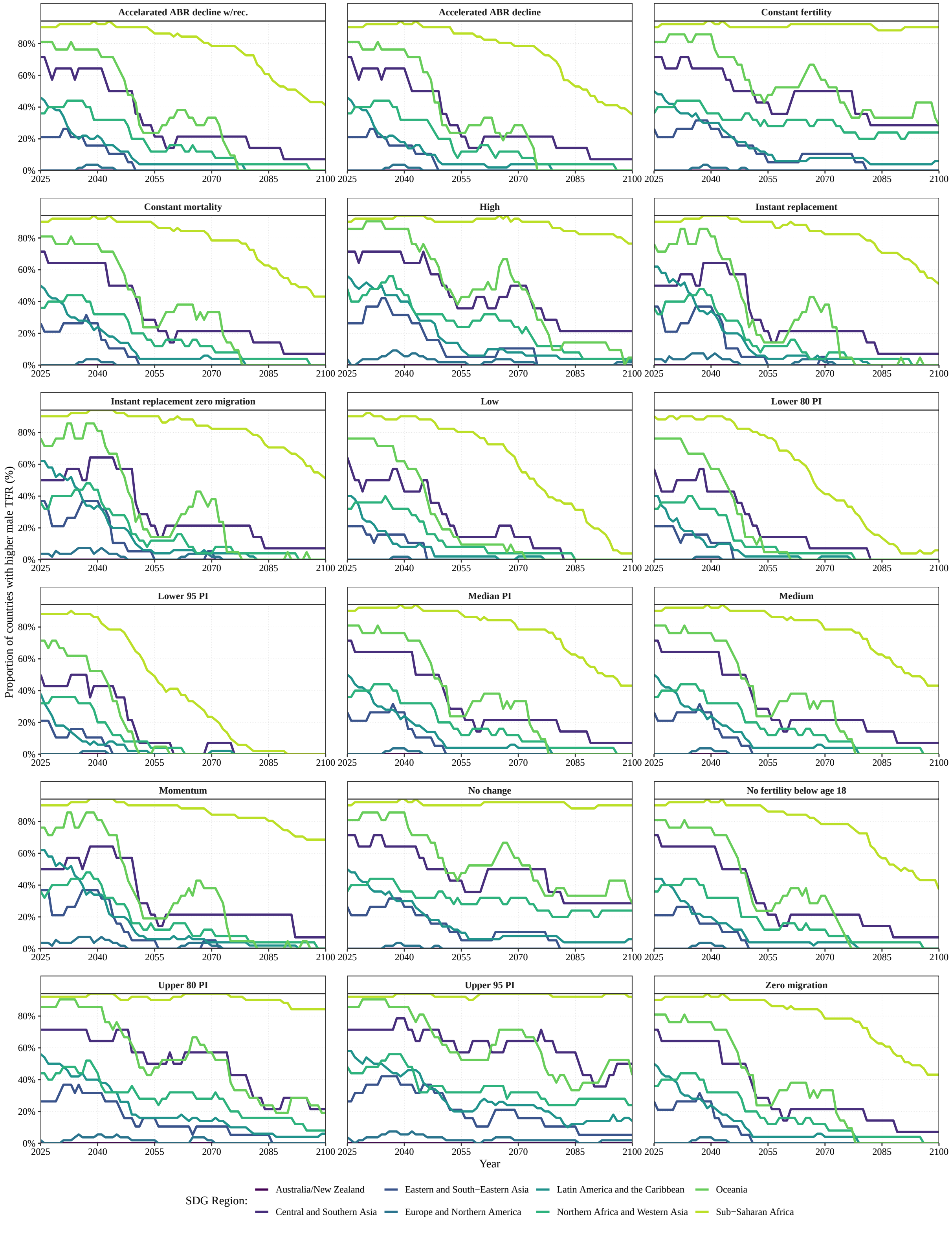

Fig. S6. This figure illustrates the share of countries and areas with higher TFR_m relative to the TFR_w in per cent (y-axis) across different WPP2024-scenarios (different panels) and geographic regions (colours) between 2025 and 2100 (x-axis).

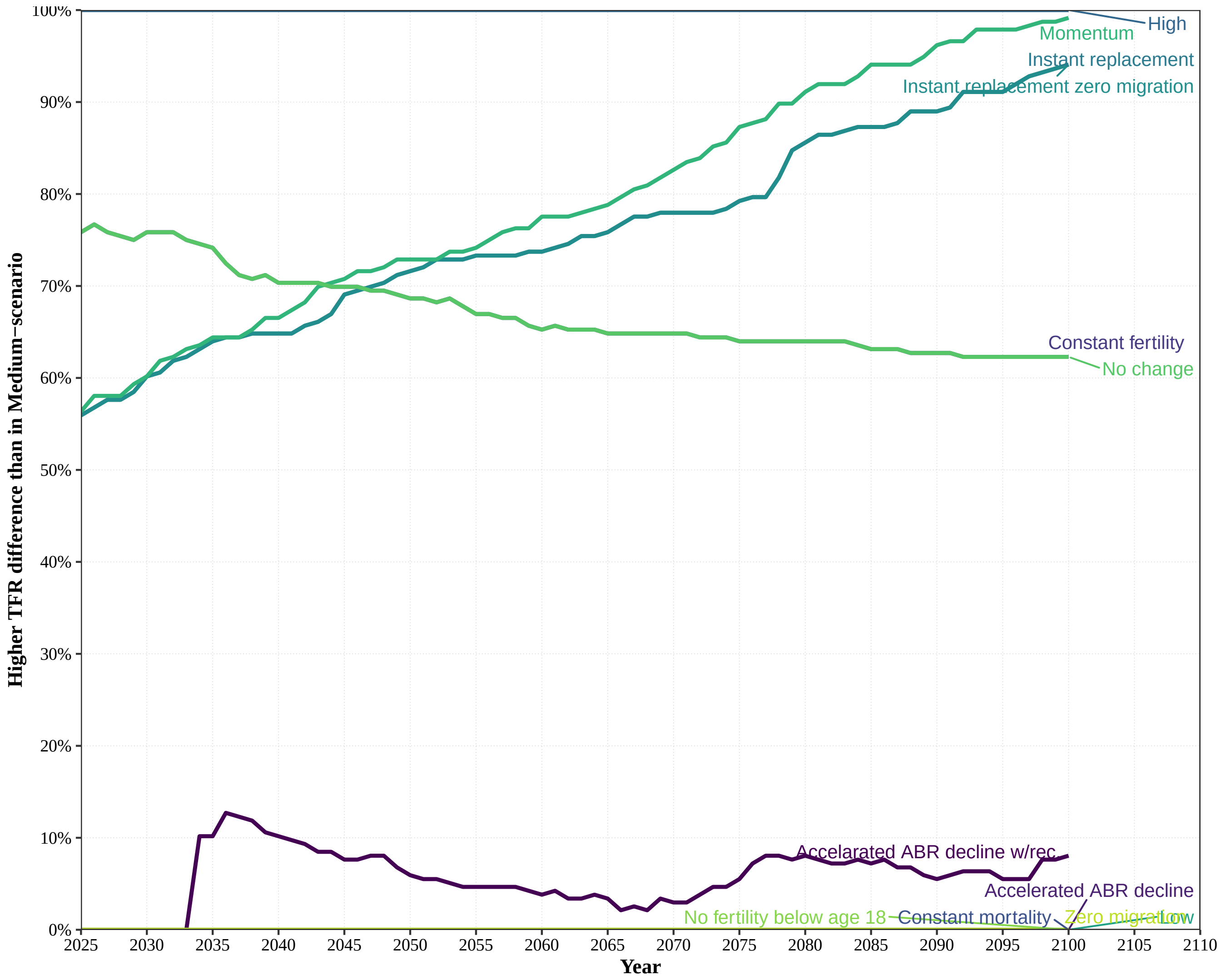

Fig. S7. This figure displays the percentage of countries and areas where the specific scenario has a higher male-female TFR difference than the medium scenario. Interpretation: the higher the share the more often the assumptions of the scenario contribute to higher TFRm relative to TFRw

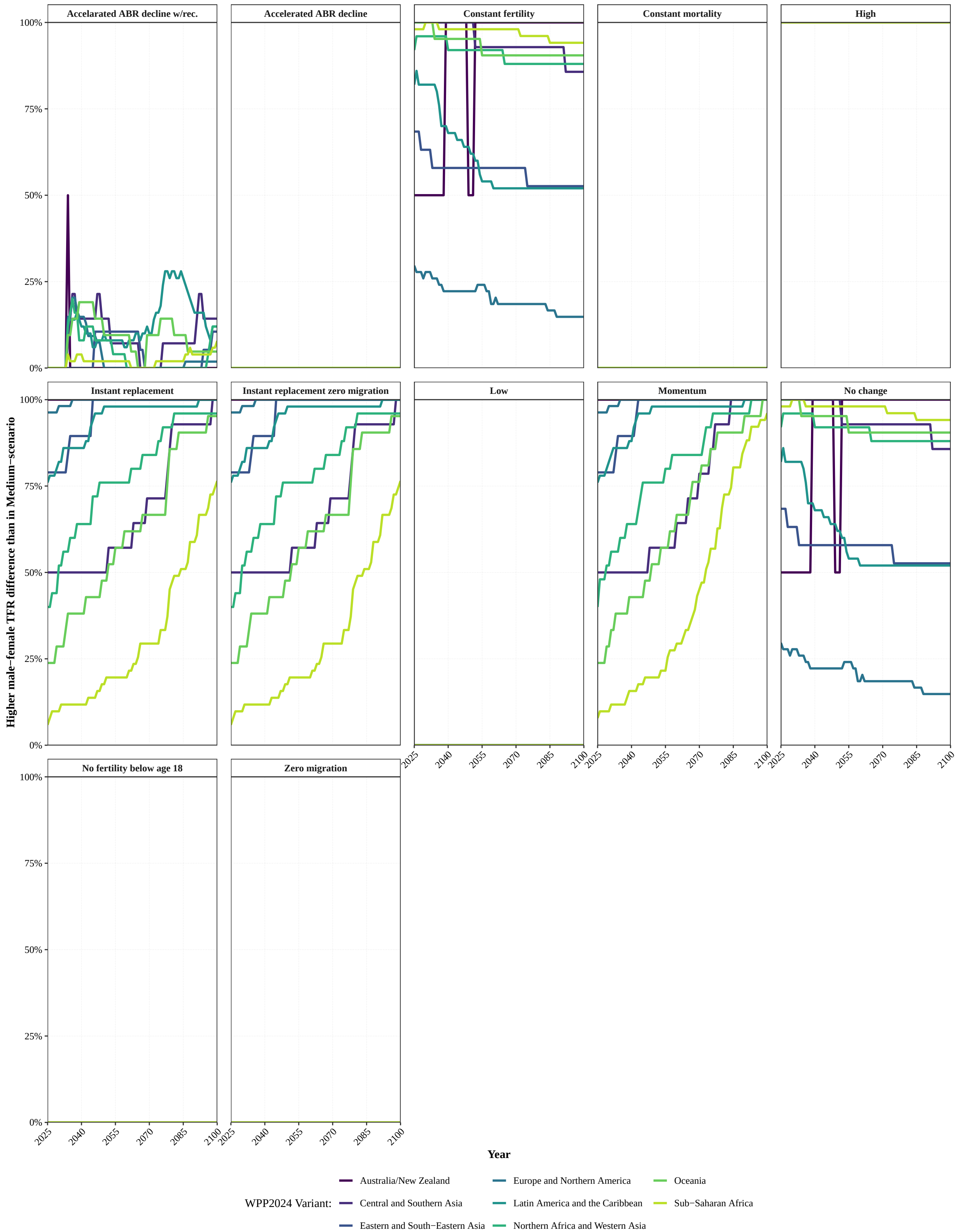

Fig. S8. This figure displays the per cent of countries and areas where the specific scenario has a higher male-female TFR difference than the medium scenario by SDG region. Interpretation: the higher the share the more often the assumptions of the scenario contribute to higher TFRm relative to TFRw

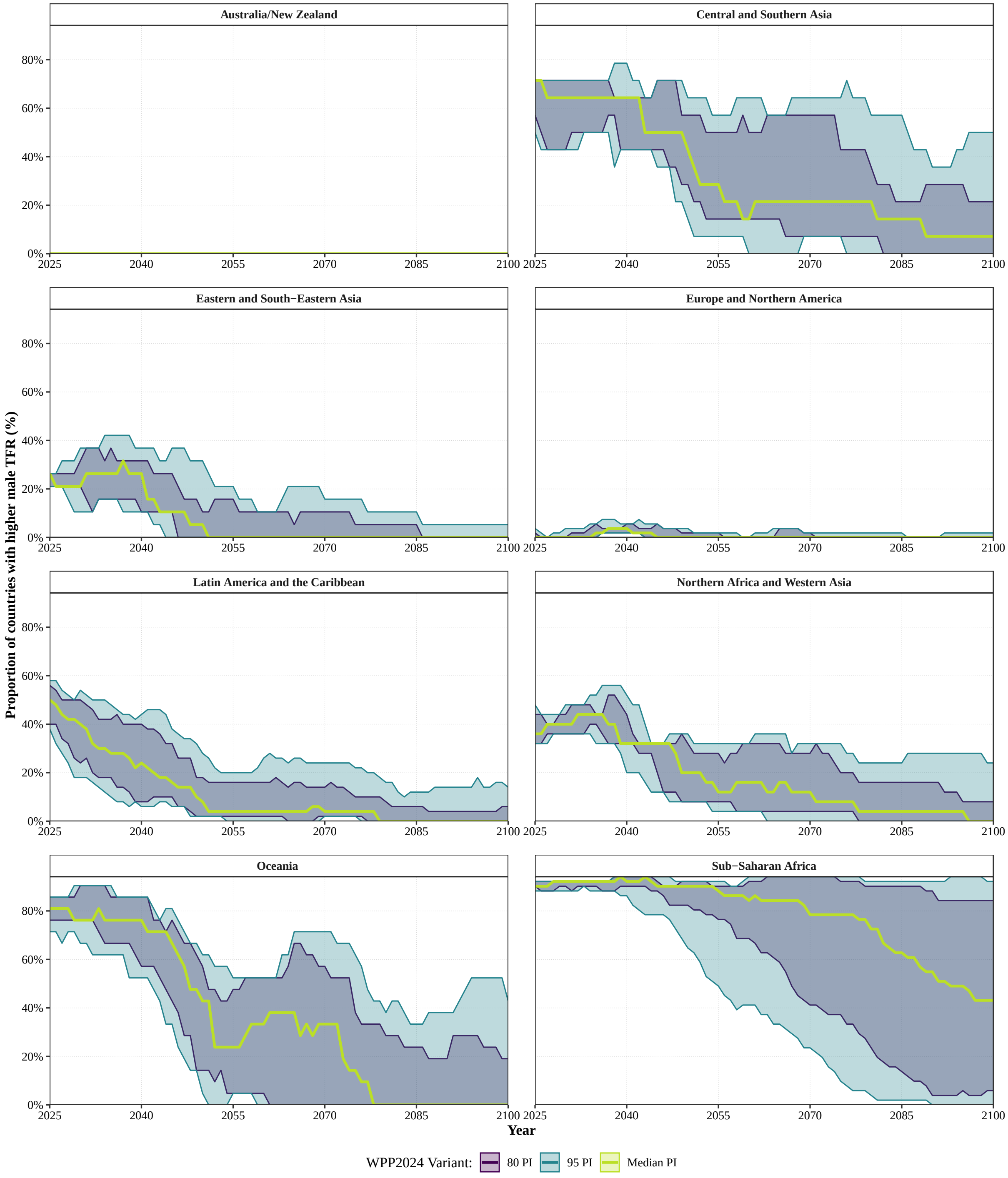

Fig. S9. This figure illustrates the share of countries and areas with higher TFR_m relative to the TFR_w in per cent (y-axis) incorporating the uncertainty in the estimation from the WPP2024 probabilistic model (different colours) and geographic regions (panels) between 2025 and 2100 (x-axis).

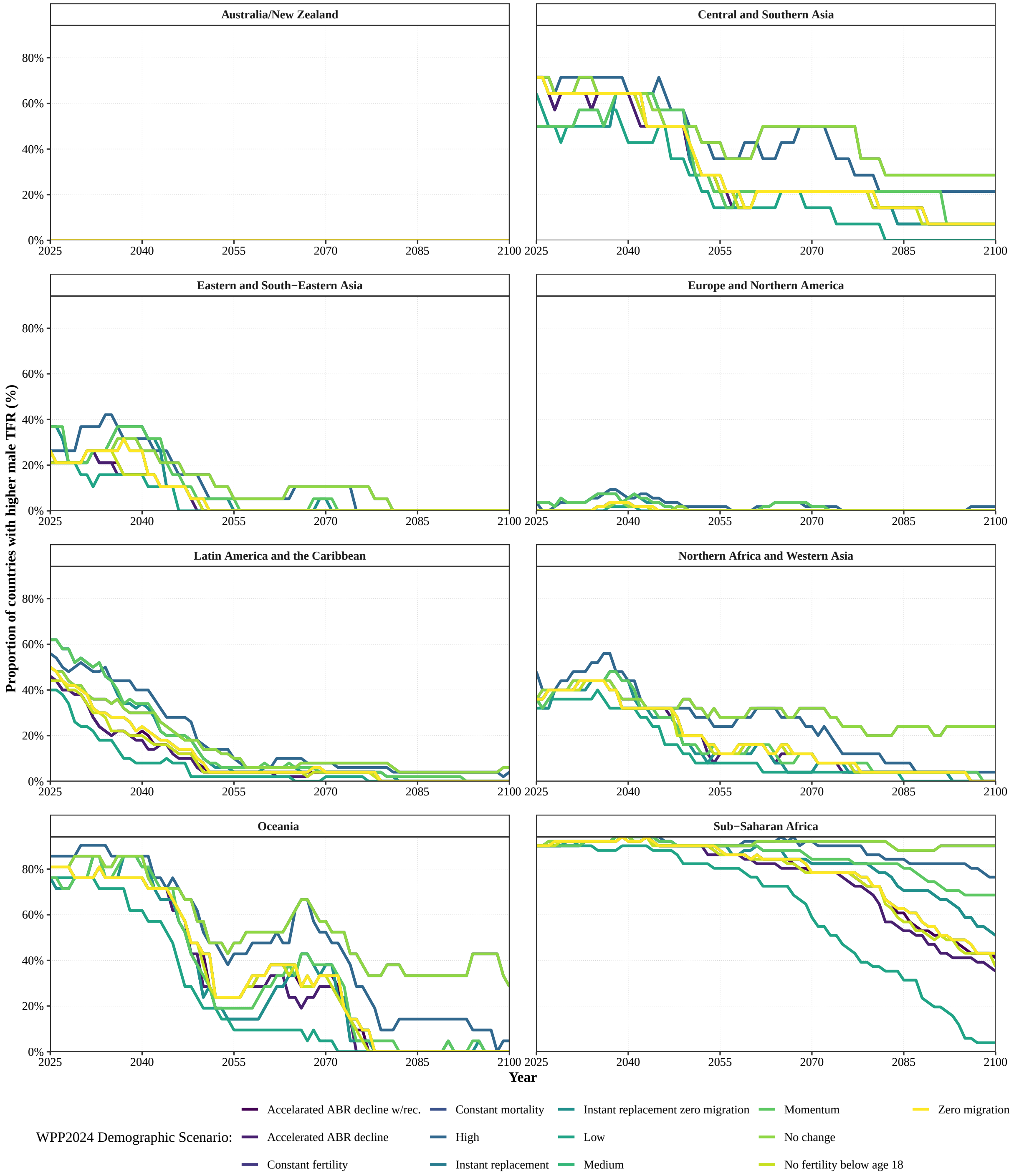

Fig. S10. This figure illustrates the share of countries and areas with higher TFR_m relative to the TFR_w in per cent (y-axis) across different WPP2024-scenarios (different colours) and geographic regions (panels) between 2025 and 2100 (x-axis).

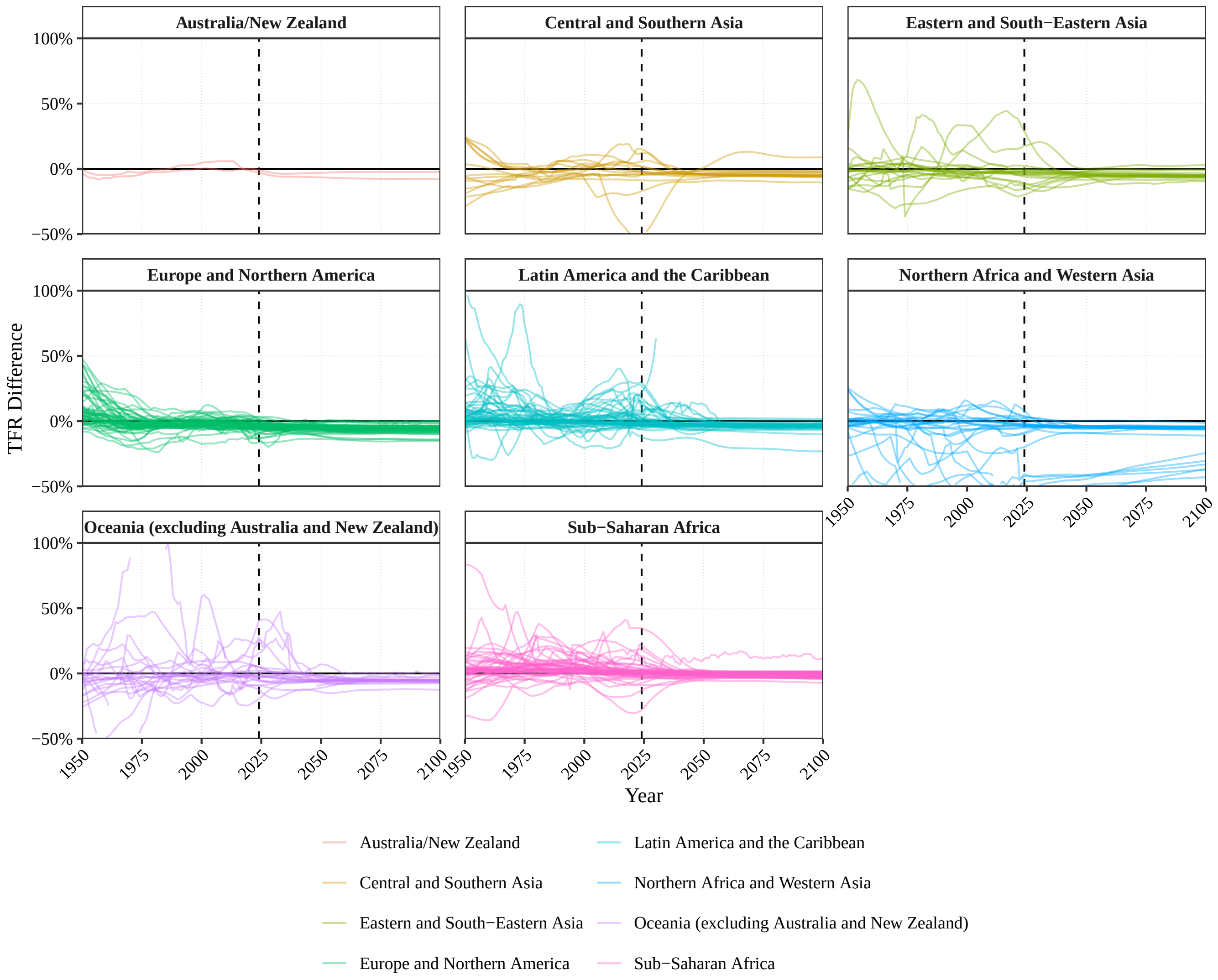

Fig. S11. Percentage difference in male to female TFR (y-axis) in the period between 1950 to 2100 (x-axis) using the standardization approach. Positive values indicate a higher TFR among men and negative values indicate a lower TFR among men. The vertical line indicates the year 2025.

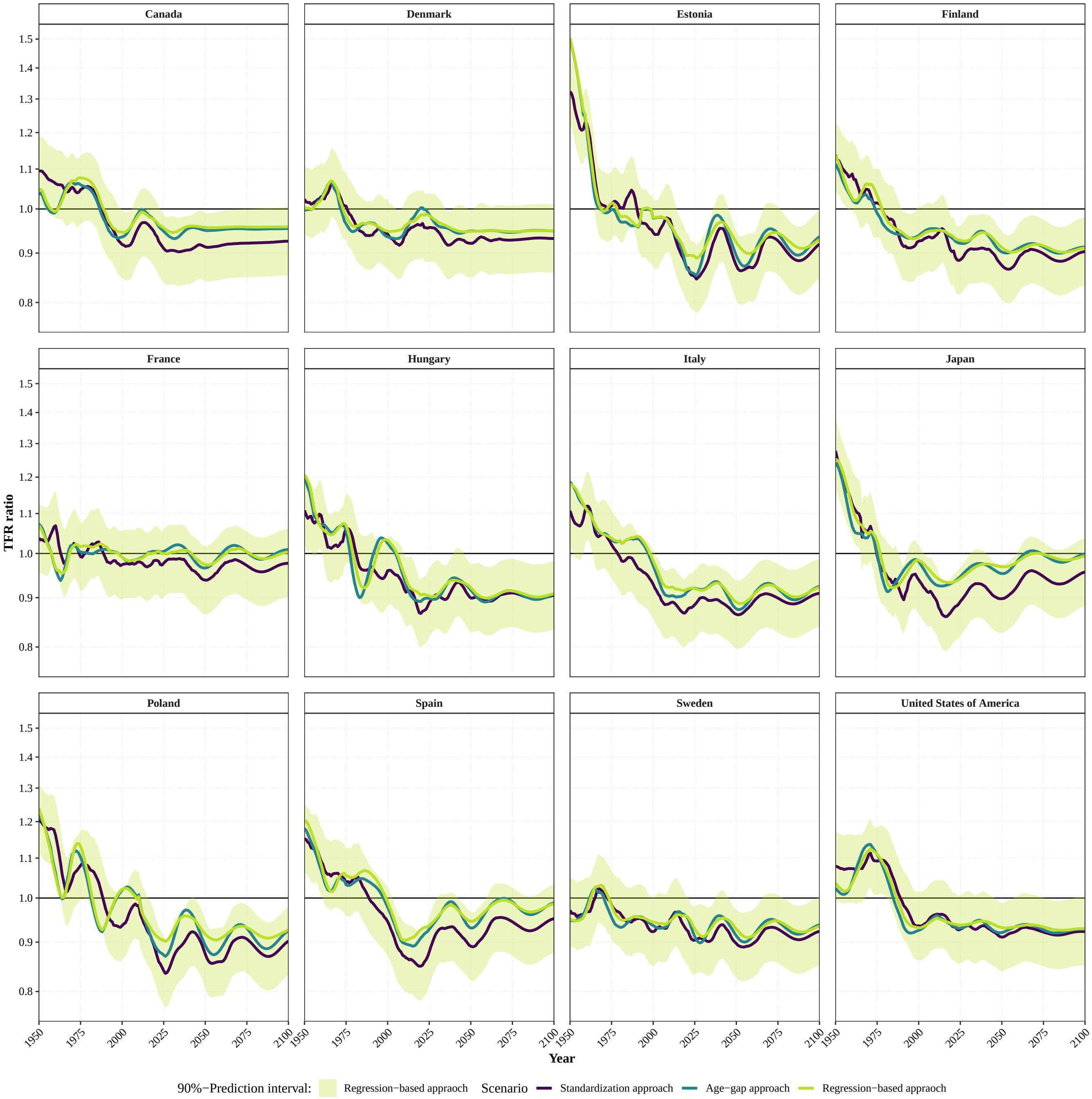

Fig. S12. This figure illustrates the impact of the estimation method on the TFR ratio ($\frac{TFR_m}{TFR_w}$) for selective countries over the period between 1950 to 2100. The purple line displays the results from the standardization method, the dark green line shows the results from the age-gap approach, and the light-green line illustrates the results from the regression-based approach including the prediction errors.

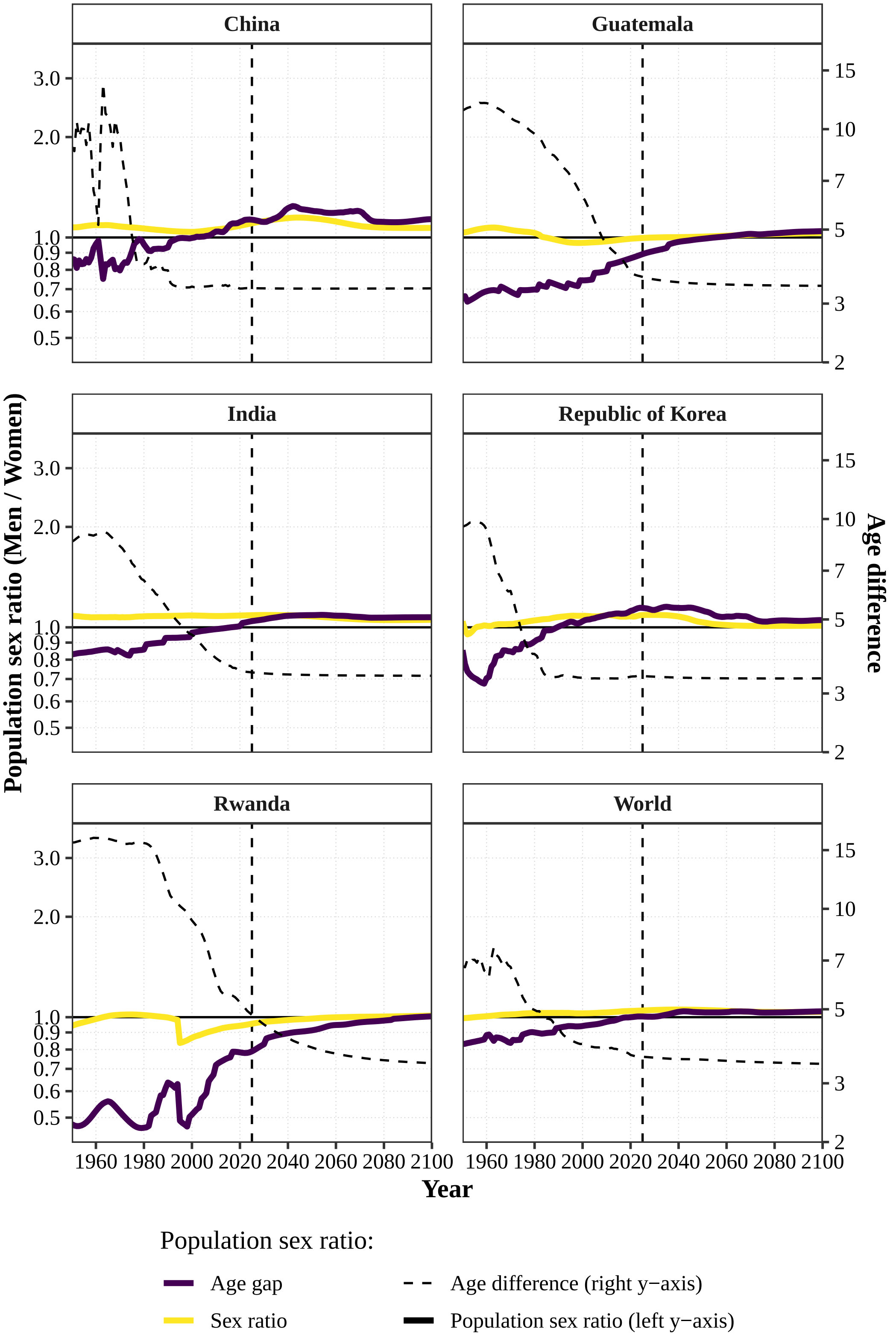

Fig. S13. Population sex ratios (y-axis) estimated with and without a changing age gap for the period between 1950 and 2100 (x-axis). The dashed line refers to the right y-axis and indicates the average age gap, which is estimated from a polynomial regression model: $age - gap = \beta_1 + \beta_2 TFR + \beta_3 TFR^2$.

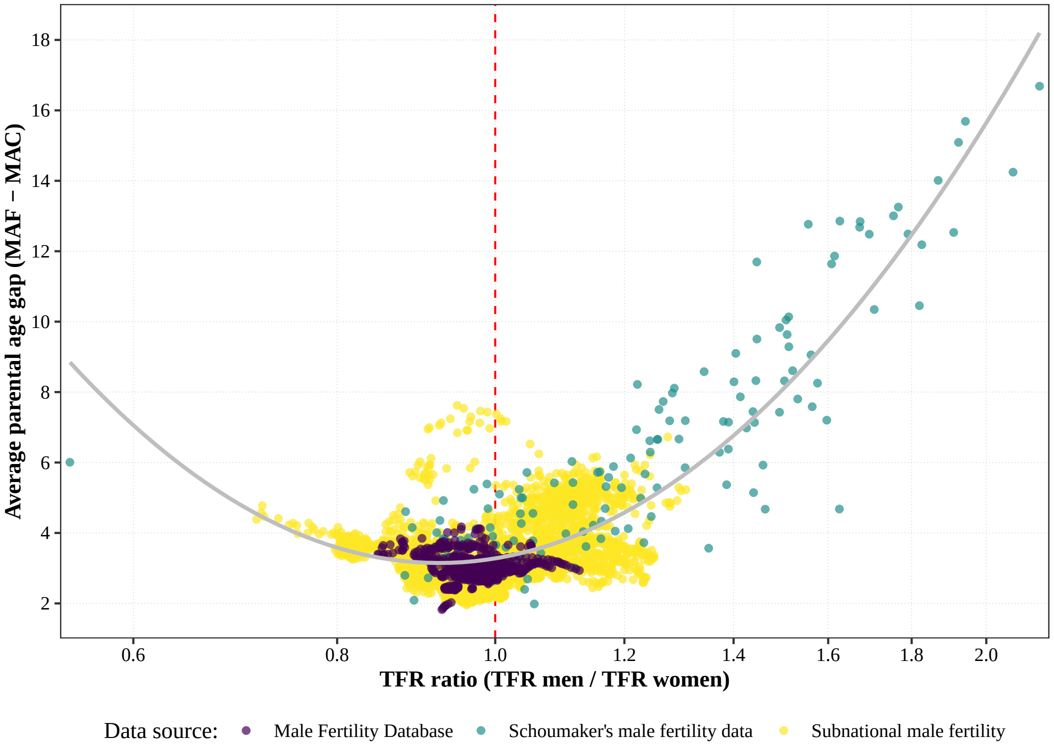

Fig. S14. Relationship between the TFR ratio ($\frac{TFR_m}{TFR_w}$) and the average parental age gap, which is the difference between the mean age of fatherhood (MAF) and the mean age at childbearing (MAC). The fitted line is a simple polynomial regression model: $y = \alpha + \beta x + \beta x^2$. Data comes from Schoumaker (7), Dudel and Klüsener (6), Max Planck Institute for Demographic Research and Vienna Institute of Demography (13), and Schubert and Dudel (5).

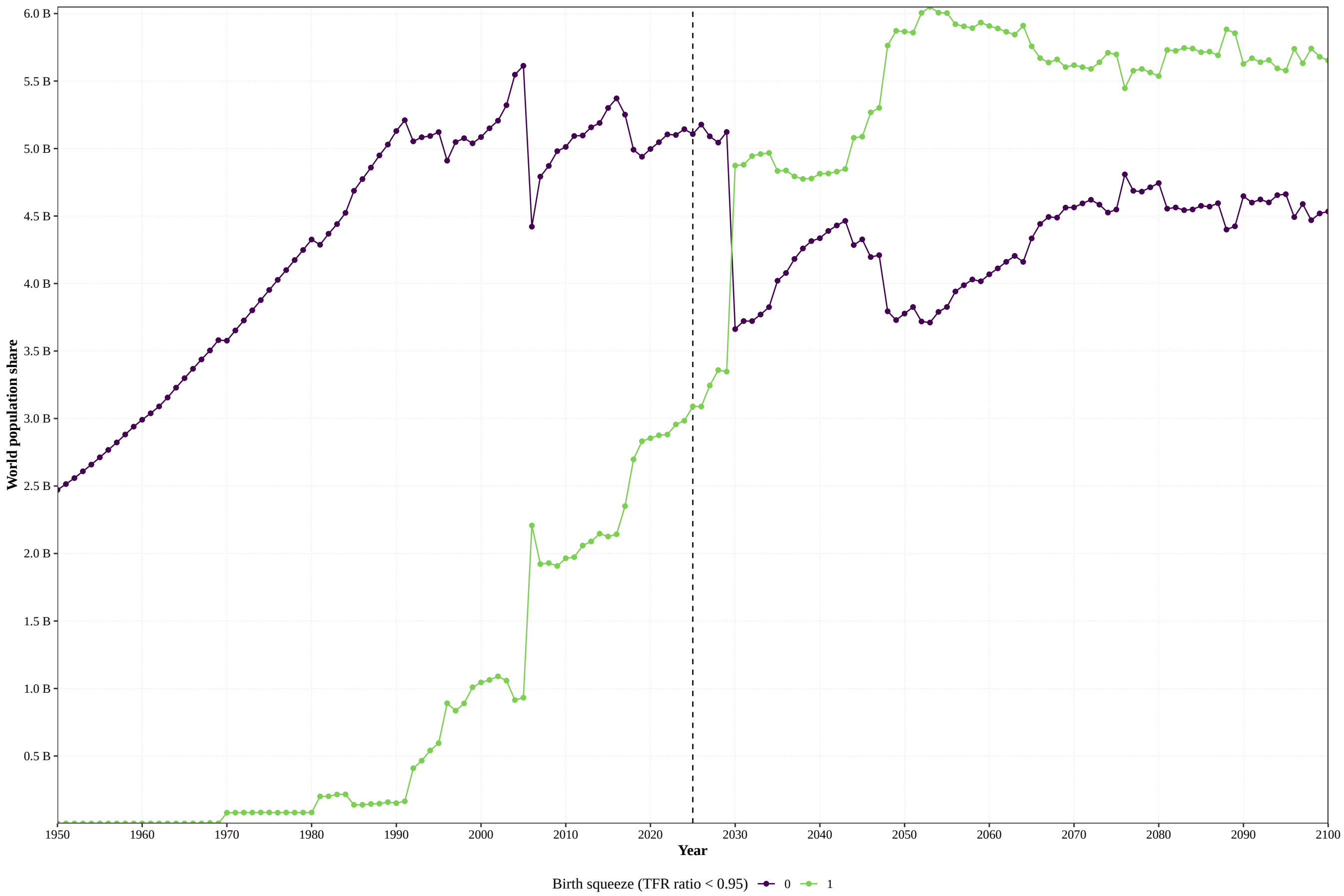

Fig. S15. This figure displays the number of people living in countries with substantially higher TFR_w than the TFR_m ($\geq 5\%$ difference) using population data from the UN WPP 2024. The figure shows that the majority of the world population will live in countries with lower TFR_m from 2030 onward.

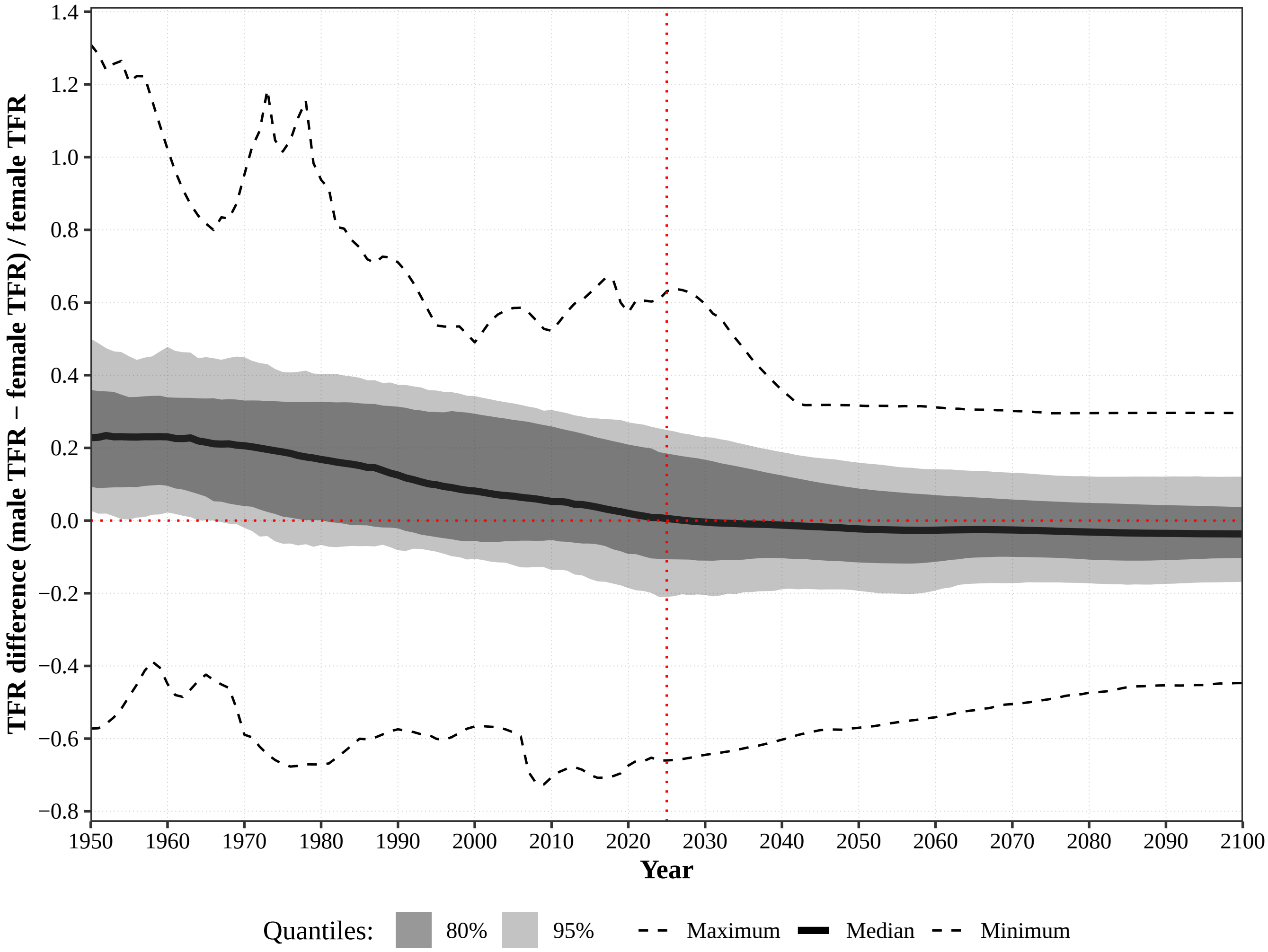

Fig. S16. Distribution of TFR difference across countries (percentage difference between TFR_m and TFR_w) across the world over time using the median, maximum, minimum, 80% quantiles, and 95% quantiles.

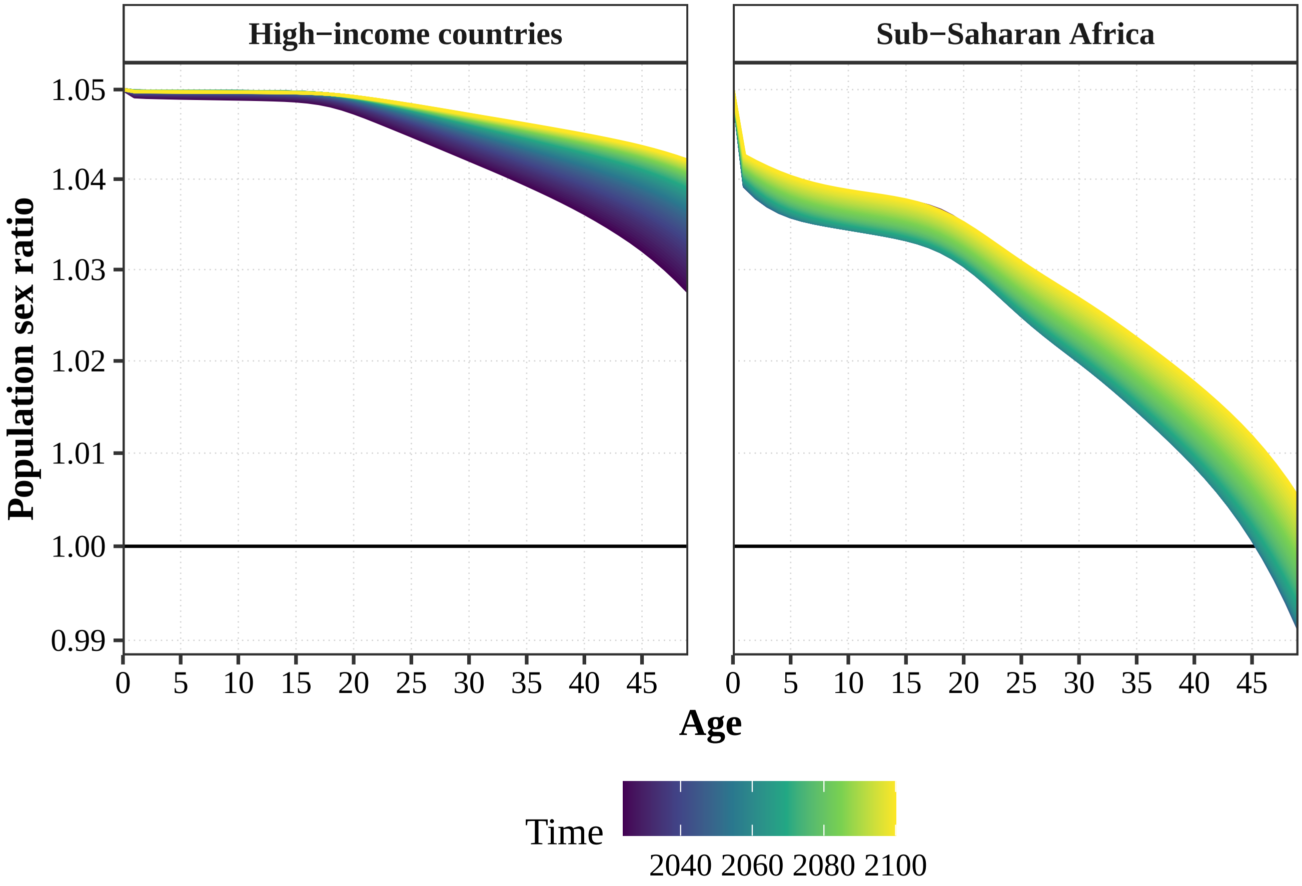

Fig. S17. This figure displays population sex ratios and the impact of changing sex ratios at birth and mortality in High-income countries vs. Sub-Sahran Africa, indicating a lasting impact of excess male mortality in Sub-Saharan Africa relative to the high-income countries.

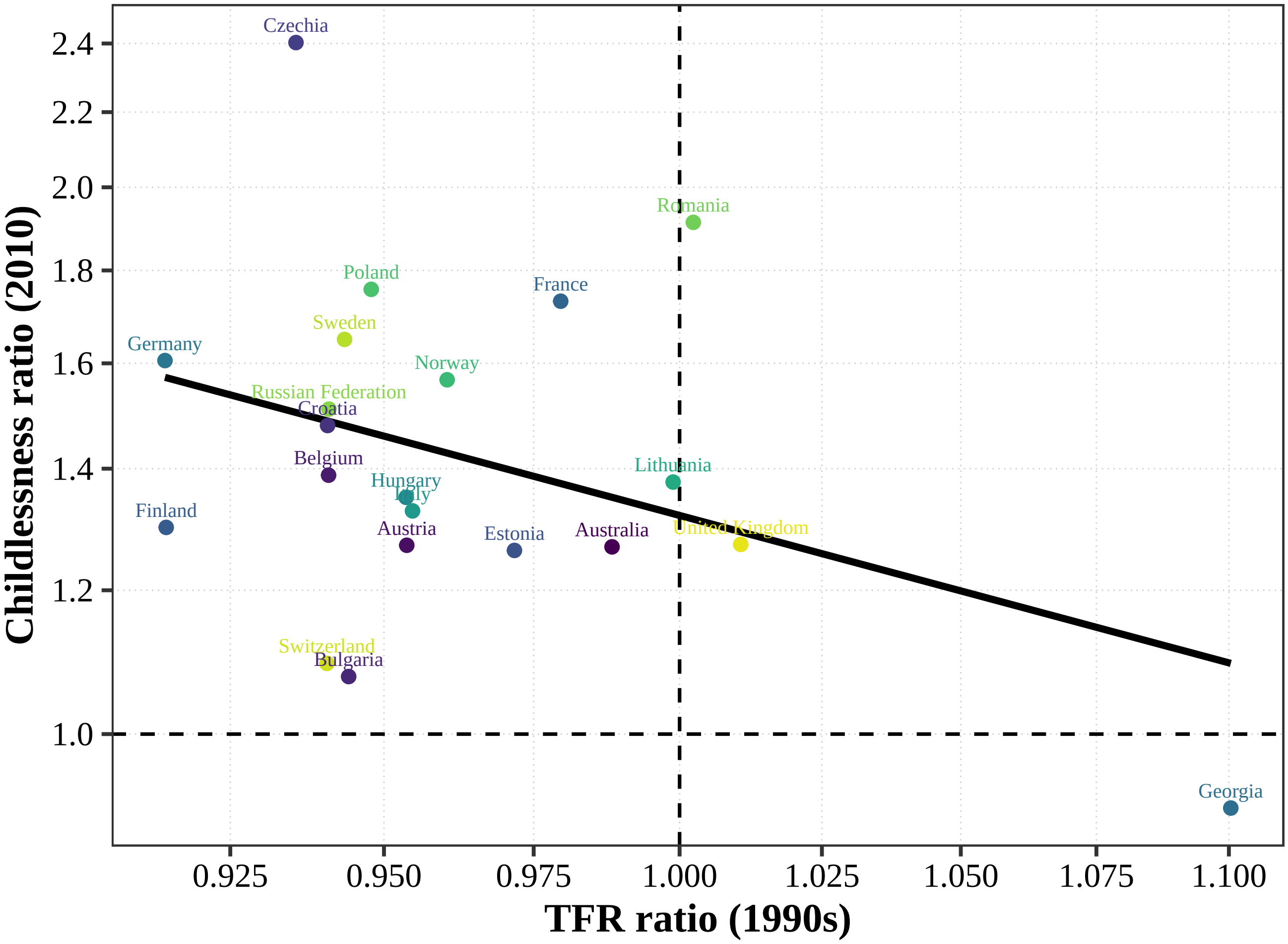

Fig. S18. This figure displays the relationship between the TFR ratio (x-axis, $\frac{TFR_m}{TFR_w}$) and the ratio of male childlessness (ages 50-55) to female childlessness (ages 45-50) using data on childlessness from Tanturri et al. (14). The black line indicates the bivariate log-log OLS regression fit.

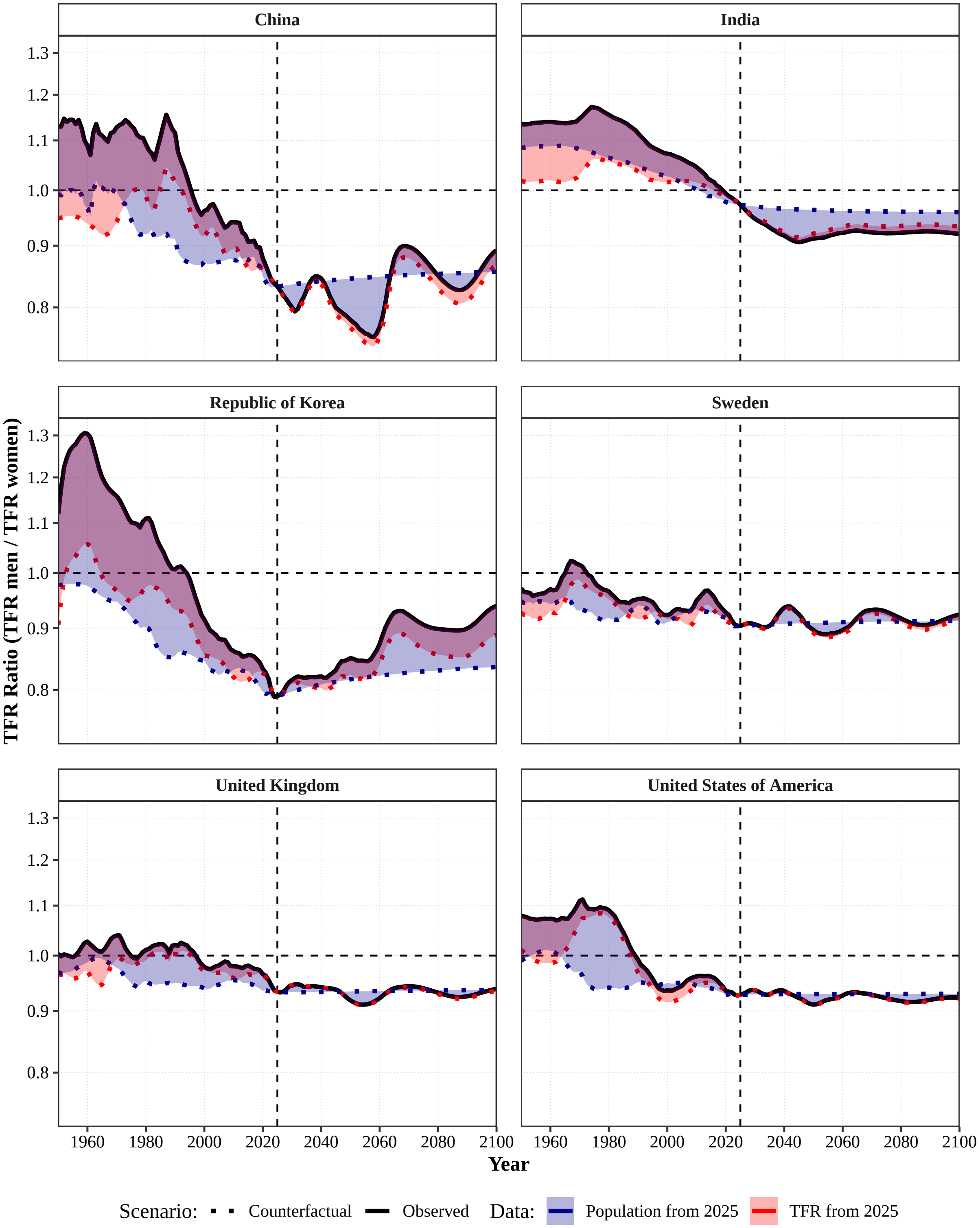

Fig. S19. Counterfactual simulation of the TFR ratio ($\frac{TFR_m}{TFR_w}$) holding either the population sex ratio and/or the TFR for women constant at the value from 2025 for the period between 1950 and 2100 (x-axis). The more the dotted line deviates from the solid line, the stronger the impact of that specific component relative to the impact in 2025.

References

1. Frances K. Goldscheider and Gayle Kaufman. Fertility and Commitment: Bringing Men Back In. *Population and Development Review*, 22:87, 1996. ISSN 00987921. .
2. Kara Joyner, H. Elizabeth Peters, Kathryn Hynes, Asia Sikora, Jamie Rubenstein Taber, and Michael S. Rendall. The Quality of Male Fertility Data in Major U.S. Surveys. *Demography*, 49(1):101–124, February 2012. ISSN 0070-3370, 1533-7790. .
3. Christian Dudel and Sebastian Klüsener. Estimating men's fertility from vital registration data with missing values. *Population Studies*, 73(3):439–449, September 2019. ISSN 0032-4728, 1477-4747. .
4. Bruno Schoumaker. Measuring male fertility rates in developing countries with Demographic and Health Surveys: An assessment of three methods. *Demographic Research*, 36:803–850, March 2017. ISSN 1435-9871. .
5. Henrik-Alexander Schubert and Christian Dudel. Subnational Birth Squeezes? Male-Female TFR Differences across Eight High- and Middle Income Countries over Time, 2025.
6. Christian Dudel and Sebastian Klüsener. Male–Female Fertility Differentials Across 17 High-Income Countries: Insights From A New Data Resource. *European Journal of Population*, 37(2):417–441, April 2021. ISSN 0168-6577, 1572-9885. .
7. Bruno Schoumaker. Male Fertility Around the World and Over Time: How Different is it from Female Fertility? *Population and Development Review*, 45(3):459–487, September 2019. ISSN 0098-7921, 1728-4457. .
8. Nico Keilman, Krzysztof Tymicki, and Vegard Skirbekk. Measures for Human Reproduction Should Be Linked to Both Men and Women. *International Journal of Population Research*, 2014(1):908385, 2014. ISSN 2090-4037. .
9. Éva Beaujouan and Tomáš Sobotka. Late Motherhood in Low-Fertility Countries: Reproductive Intentions, Trends and Consequences. In Dominic Stamp, editor, *Preventing Age Related Fertility Loss*, pages 11–29. Springer, 2017.
10. Christian Dudel, Yen-hsin Alice Cheng, and Sebastian Klüsener. Shifting Parental Age Differences in High-Income Countries: Insights and Implications. *Population and Development Review*, 49(4):879–908, 2023. ISSN 1728-4457. .
11. Ludwig Fahrmeir, Thomas Kneib, Stefan Lang, and Brian D. Marx. *Regression: Models, Methods and Applications*. Springer Berlin Heidelberg, Berlin, Heidelberg, 2021. ISBN 978-3-662-63881-1 978-3-662-63882-8. .
12. United Nations Department of Economic and Social Affairs. World Population Prospects 2024: Methodology of the United Nations population estimates and projections. Technical report, United Nations, Department of Economics and Social Affairs, Population Division, 2024.
13. Max Planck Institute for Demographic Research and Vienna Institute of Demography. Human Fertility Database (HFD). Technical report, Max Planck Institute for Demographic Research and Vienna Institute of Demography, 2014. URL www.humanfertility.org.
14. Maria Letizia Tanturri, Melinda Mills, Anna Rotkirch, Tomáš Sobotka, Judit Takásc, Anneli Miettinen, Cristina Faludi, Venetia Kantsa, and Despina Nasiri. State-of-the-art report. Childlessness in Europe. Technical Report 32, Families and Societies, 2015.
15. Henrik-Alexander Schubert and Christian Dudel. Too many men? Subnational population imbalances and men's childlessness in Finland. *Population Studies*, 0:1–21, 2025. ISSN 0032-4728. .
